# Characterization and clustering of immunocompetent proteins based on ESM-2 sequence embedding and physicochemical feature fusion

**DOI:** 10.3389/fimmu.2026.1918918

**Published:** 2026-09-30

**Authors:** Xingxing Liu, Rui Zhang, Jie Li, Li Zhang, Panpan Tong, Jinxin Xie

**Affiliations:** 1 School of Xinjiang Second Medical College, Xinjiang, Karamay, China; 2 Laboratory of Animal Etiology and Epidemiology, College of Veterinary Medicine, Xinjiang Agricultural University, Urumqi, China

**Keywords:** antihistamine protein, cross-species verification, ESM-2 protein language model, multi-feature fusion, physicochemical characteristics of protein, protein cluster analysis

## Abstract

**Objective:**

Traditional sequence-comparison methods face challenges when classifying and identifying low-homology proteins formed by convergent evolution. In this exploratory study, we built a PCA-based unsupervised clustering workflow combining high-dimensional embeddings from the frozen pre-trained protein-language model ESM-2 and weighted-fused physicochemical features. The workflow was explored for family-level grouping, identification of putative functional conservation, and cross-species evolutionary analysis of immune-active proteins. An independent external test dataset was further used to preliminarily probe its potential generalization behaviour.

**Methods:**

Nineteen arthropod-derived immunocompetent proteins (tick lipocalin antihistamine proteins, mosquito D7 salivary proteins, dust mite allergens, and two additional arthropod immune-active proteins) formed our reference dataset. Eleven test sequences, comprising nine independent vertebrate Cathelicidin peptides and two tick lipocalin positive controls, including nine vertebrate cathelicidin antimicrobial peptides and two tick lipocalin homologs, served as the external test dataset. Using Python3.9, we implemented an analytical pipeline. The ESM-2 t33_650M_UR50D model in frozen pre-trained mode generated 1280-dimensional sequence embeddings. Six core physicochemical and secondary-structure descriptors were computed. An empirically-determined weight ratio (0.7:0.3) was applied to build a weighted fused dissimilarity matrix. After full feature concatenation, PCA-based clustering and visualization were performed.

**Results:**

On the reference data set, the fusion features showed a significant visual separation trend among different functional protein families. The fusion dissimilarity value within the family is usually lower than the dissimilarity value between the families, and there is a statistically significant difference at the group level (P < 0.05). The 9 vertebrate cathelicidin sequences in the test dataset form a unique cluster separated from the arthropod reference protein. The observed clustering patterns are generally consistent with the evolutionary relationship of species and the calculated physical and chemical properties.

**Conclusion:**

This exploratory ESM-2-embedding and physicochemical-feature-fusion PCA clustering workflow shows promising descriptive performance for immune-active proteins. It can generate family-level grouping without relying on high-quality multiple-sequence alignments and may help analyse low-homology protein datasets. Nevertheless, given dataset-size limitations and the absence of quantitative clustering-validation metrics, its broad-scale classification accuracy, stability and generalizability remain to be further validated on larger datasets. This pipeline provides a reproducible bioinformatic framework for exploratory family screening, evolutionary interpretation and functional hypothesis generation for animal-derived immune-active proteins.

## Introduction

Protein serve as the core functional carriers of life activities, and its functional conservation represents a key molecular basis for maintaining adaptive evolution and physiological homeostasis in species (1–3). Over the course of long-term natural selection and species divergence, homologous functional proteins across different species—and even among various developmental stages of the same species—typically exhibit evolutionary characteristics indicative of sequence heterogeneous differentiation and biological function convergence (4). During long-term natural selection and species differentiation, some proteins exhibit sequence divergence at the primary-sequence level while retaining functional conservation. Such phenomenon can occur among true homologous proteins derived from a common ancestor, as well as among convergently-evolved functionally-analogous proteins without shared ancestry (5–7). Although these functional homologous proteins often display low levels of amino acid sequence homology, they can execute highly consistent biological effects at the physiological level (8). A typical example is the tick-derived antihistamine functional protein: homologous antihistamine proteins encoded by genes from different tick species and differentially expressed across diverse developmental stages demonstrate significant differentiation at the primary sequence level, yet they can stably and specifically bind to the host histamine (9, 10). This interaction achieves the biological effect of inhibiting the host local inflammatory response and immune defense. Such molecular characteristics present significant challenges for traditional sequence homology comparison methods in screening and functionally comparing homologous functional proteins (9).

The rapid advancement of artificial intelligence and bioinformatics has led to the emergence of Protein Language Models (PLMs) (11, 12), which provide a complementary way for traditional sequence-based methods by taking advantage of the unsupervised pre-training advantages brought by massive natural protein sequences. These models provide a novel research paradigm for mining higher-order functional features of proteins. Notably, Evolutionary Scale Modeling (ESM) (13, 14) capitalizes on pre-training weights to explore amino acid sequence arrangement patterns, functionally conserved domain information, and implicit spatial structure features, effectively transforming disordered protein sequences into standardized high-dimensional functional embedding vectors. This approach has found widespread application in areas such as protein function prediction, family classification, and molecular evolution analysis. In biology, the recurrence of similar functional features across evolutionary lines is significant (15), as it allows for the decoding of relationships between genomes or protein sequences and specific functions (16).

Nevertheless, systematic challenges remain in the comparative study of analogous functional proteins using PLMs. Most existing research solely relies on embedded features for qualitative clustering visualization, without establishing a standardized quantitative evaluation system for functional dissimilarity (17). Moreover, a lack of statistical significance verification regarding group differences results in a high level of subjectivity in analysis outcomes and diminishes the reliability of experimental conclusions. Furthermore, prevalent methods for discriminating convergent evolutionary proteins are overly dependent on phylogenetic tree construction and neutral evolutionary sequence simulation. Consequently, the universality and applicability of these methods are limited, making it difficult to conduct comparative analyses of distant homologous functional proteins that lack clear phylogenetic relationships.

In the realm of parasite-host interactions and vertebrate innate immune regulation, arthropod salivary gland secretory active proteins and vertebrate endogenous antimicrobial active peptides represent two classical molecular groups with distinct evolutionary origins and functional differentiation. Proteins from the lipocalin family (18), D7 long-chain salivary proteins (19), and histamine-binding proteins (20) secreted by blood-sucking arthropods are pivotal effectors for ticks, mosquitoes, and blood-sucking bedbugs, facilitating host immunosuppression, neutralizing inflammatory mediators such as histamine, and enabling continuous blood-feeding behavior. Conversely, Cathelicidin family cationic antimicrobial peptides (21, 22), which are widely expressed across vertebrates, serve as key functional peptides within the body’s natural immune defense pathways. These peptides exhibit multiple biological activities, including broad-spectrum inhibition of pathogenic microbes, regulation of inflammatory signals, and remodeling of immune homeostasis.

This study selected 19 immune-active proteins, encompassing tick-derived antihistamine proteins, mosquito salivary functional proteins and mite allergenic proteins, to establish a reference dataset. An empirically selected weight ratio (0.7:0.3) was used for feature fusion, with sensitivity analysis performed across seven configurations. Additionally, 11 independent exogenous sequences were identified to form a test validation dataset, featuring 9 cross-species Cathelicidin antimicrobial peptide sequences from Aves, Amphibia, Reptilia, and Mammalia, as well as 2 tick-derived Lipocalin homologous protein sequences. Utilizing Python 3.9, an automated analysis system was constructed, integrating tools such as PyTorch, ESM-2, Biopython, NumPy, SciPy, Scikit-learn, Pandas, Matplotlib, and Seaborn. This system facilitated sequence reading, physical and chemical calculations, high-dimensional embedding extraction, feature fusion, PCA dimensionality reduction, dissimilarity matrix construction, and visual representation. The coding operations exhibit strong reproducibility and high stability, effectively addressing research needs in functional protein classification, family identification, and cross-species verification. Moreover, a horizontal efficiency comparison with traditional sequence homology alignment analysis was conducted to elucidate the technical advantages and application scenarios of the fusion feature clustering analysis system. This initiative aims to provide new analytical tools and systematic research approaches for the rapid screening of distant homologous proteins, analysis of biological convergence evolution mechanisms, accurate annotation of protein functions, and high-throughput screening of medicinal active peptides.

## Materials and methods

### Construction of protein sequence data set

#### Reference dataset

This study is an exploratory unsupervised analysis. A total of 19 immunocompetent protein sequences from arthropods with verified functions were collected to construct a reference data set. All the original sequences were retrieved from the NCBI public database and strictly preprocessed to eliminate invalid entries, including sequences containing ambiguous residues, incomplete fragments, and redundant repeats. The qualified sequences were uniformly converted into a standard FASTA format to ensure the data quality of subsequent feature extraction. Since the ESM-2 model is used in a frozen pre-training state without retraining or parameter updating, these 19 sequences are only used for feature embedding generation, multi-dimensional feature fusion, and preliminary determination of clustering boundaries. In order to avoid loop verification and methodological overfitting, the clustering threshold is not artificially adjusted to optimize the clustering performance of the reference data set. The rationality and clustering stability of family separation are further independently verified by a completely mutually exclusive external test set, rather than relying solely on reference data. Detailed classifications and species sources are presented in **Supplementary Table S1**.

#### Independent test dataset

The test set comprised 11 sequences: nine vertebrate Cathelicidin antimicrobial peptides as independent cross-phylum validation samples, plus two tick lipocalin sequences included as positive controls to verify projection stability. These samples were not used to fit the StandardScaler or PCA loadings or feature space optimization, and are only used to evaluate the generalization performance and clustering stability of our unsupervised feature fusion pipeline. These sequences were categorized into two functional groups (**Supplementary Table S2**):

Cathelicidin antimicrobial peptides from vertebrates (9): Homologous Cathelicidin antimicrobial peptide sequences were selected from various evolutionary levels, including birds (*Coturnix coturnix, Phasianus colchicus*), amphibians (*Quasipaa spinosa, Leptobrachium liui, Hoplobatrachus rugulosus*), reptiles (*Crocodylus siamensis, Python bivittatus*), and mammals (*Saguinus oedipus,Bubalus bubalis*). This selection aimed to explore the distribution and differentiation characteristics of cross-species homologous functional proteins within the fused feature space.

Tick-derived Lipocalin antihistamine proteins (2): Homologous sequences from *Ixodes ricinus* and *Argas monolakensis* were included, exhibiting consistent biological functions and evolutionary attributes with the same family proteins in the reference-set proteins, thus probing the workflow’s capacity for family-level grouping of known functional-family proteins.

#### ESM-2 high-dimensional sequence embedding feature extraction

The ESM-2 t33_650M_UR50D pre-trained model was used to generate high-dimensional embedding representations for protein sequences. Embeddings derived from this transformer model may capture latent signals associated with evolutionary conservation, structural constraints and potential functional patterns of amino-acid sequences.

#### Quantitative calculation of physical and chemical characteristics of protein

Using Biopython-based workflows, six core physicochemical and secondary-structural indicators closely tied to the biological functions of each experimental protein sequence were measured in batches: molecular weight (MW), theoretical isoelectric point (pI), grand average of hydropathicity (GRAVY), net charge at pH7.0, α-helix content (Helix, %), and β-sheet content (Sheet, %). These indicators were structured into a comprehensive data table. Sample charts and Python code can be found in **Supplementary Files 2–4** for subsequent feature fusion and dissimilarity calculations.

#### Multi-dimensional feature standardization preprocessing

Due to the substantial differences between the embedded features of the ESM high-dimensional sequence and the basic physical and chemical features of the proteins—particularly regarding numerical intervals and data dimensions—direct feature fusion often leads to imbalances in feature weight proportions. Therefore, the Z-score standardization method was employed to standardize the two types of features independently. The specific formula for this preprocessing step is as follows:


$$X_{norm}=\frac{X-\mu }{\sigma }$$

In this formula, Xnorm is the normalized eigenvalue, X is the original eigenvalue, μ is the mean of the feature dimension, and σ is the standard deviation of the feature dimension.

After Z-score standardization, the mean value of these two sets of features should be 0, and the standard deviation should be 1.This process eliminated the impact of dimensional differences and numerical interval discrepancies on subsequent feature fusion and dissimilarity calculations, thereby ensuring the scientific rigor and stability of the fused features.

#### Two-dimensional dissimilarity matrix construction and multi-feature weighted fusion strategy

Utilizing the standardized sequence embedding features and physicochemical characteristics, both the cosine dissimilarity matrix and the Euclidean distance dissimilarity matrix between samples were constructed. By considering the evolutionary patterns and functional expression characteristics of immune-active proteins, the empirically selected feature fusion weight ratio was determined, resulting in the development of the composite fusion dissimilarity matrix. The specific steps are outlined as follows:

Sequence embedding dissimilarity matrix (S_emb_): Using standardized high-dimensional ESM-2 embedding features, cosine distance was applied to calculate the embedding-level dissimilarity between sequences. The cosine-distance value ranges from [0,2], where values closer to 0 indicate higher functional-feature similarity between samples. The specific formula used is as follows:


$$S_{emb}(i,j)=1-\cos(\theta )=1-\frac{{\vec{E}}_{i}\cdot {\vec{E}}_{j}}{∥{\vec{E}}_{i}∥\times ∥{\vec{E}}_{j}∥}$$

In this formula, ${\vec{E}}_{i}$ and ${\vec{E}}_{j}$ are the standardized ESM-2 embedding vectors of sample *i* and sample *j*, respectively.

Physicochemical-derived dissimilarity matrix ( ${\text{D}}_{\text{phy}}$): Based on the six standardized physicochemical characteristics, Euclidean distance *d_ij_* was computed to quantify pairwise physicochemical divergence. First, Euclidean distance was converted into similarity via reciprocal transformation $S_{phy}(i,j)=\frac{1}{1+d_{ij}}$.To obtain a dissimilarity metric consistent with cosine distance, we further transformed similarity into dissimilarity:


$${\text{D}}_{\text{phy}}(i,j)=1-S_{phy}(i,j)=1-\frac{1}{1+d_{ij}}$$

In this formula, *d_ij_* represents the Euclidean distance between the standardized physicochemical characteristics of sample *i* and sample *j*. Larger ${\text{ D}}_{\text{phy}}$ values indicate greater physicochemical dissimilarity between samples.

Prior to weighted fusion, each dissimilarity matrix was independently rescaled to the unified interval [0,1] using min-max normalization. This scale-harmonization step ensures that the weighting coefficients (0.7 and 0.3) carry interpretable meaning. After normalization, higher values in both matrices represent greater dissimilarity.

Weighted fusion dissimilarity matrix (F): Empirically-set weight coefficients of 0.7 for sequence-embedding dissimilarity and 0.3 for physicochemical dissimilarity were adopted. The final composite fused dissimilarity matrix was constructed by weighted summation of the min-max-normalized dissimilarity matrices. The specific calculation formula for the fusion dissimilarity matrix is:


$$\text{F}(i,j)=0.7{\text{S}}_{\text{emb}\_\text{norm}}(i,j)+0.3{\text{D}}_{\text{phy}\_\text{norm}}(i,j)$$

Larger values of F (i,j) correspond to greater overall feature dissimilarity between two protein samples.

The resulting fused dissimilarity matrix for all samples was organized into structured data tables. Sample charts and the corresponding Python code are made available in **Supplementary Files 3, 4** for subsequent cluster analysis and visualization.

#### Sensitivity analysis and ablation analysis

In order to evaluate the effect of modal weighting on family separation, seven weighting configurations were compared: only using ESM-2 embedding (1.0: 0), only using physical and chemical descriptors (0: 1.0), and five mixing ratios (0.9: 0.1,0.8: 0.2,0.7: 0.3,0.6: 0.4,0.5: 0.5). The ratio of the average difference between families to the average difference within the family (inter-family/intra-family ratio) was used as a quantitative separation index. The highest cross-family/intra-family ratio (1.88) was obtained using the ESM-2 embedding method only, while the lowest value (1.10) was obtained using the physicochemical descriptors only. The configuration of 0.7: 0.3 maintained a good ratio of 1.53, and achieved a balance between discriminant ability and physicochemical interpretability, so it was selected as the main analysis weight.

#### Principal component analysis

To visualize the fused feature space, principal component analysis (PCA) was performed on the weighted concatenated feature matrix. Specifically, the standardized ESM-2 embedding matrix (1280 dimensions) and the standardized physicochemical descriptor matrix (6 dimensions) were multiplied by their respective fusion weights (0.7 and 0.3) prior to horizontal concatenation, ensuring that the PCA input was consistent with the weighted fusion scheme used for dissimilarity matrix construction. PCA was then applied to the resulting 1286-dimensional weighted feature matrix, and the first two principal components were retained for visualization. The proportion of variance explained by each principal component was recorded.

For independent validation, all standardized parameters (mean and standard deviation of StandardScaler) and PCA loads were fitted using only 19 reference proteins. The 11 test set proteins were then projected into the feature space of the frozen reference set using only ‘ transform ‘, without re-fitting scaler or PCA. This ensures that the test sample does not participate in the estimation of the normalized parameters or the PCA axis, and maintains the independence of the verification. By superimposing the projected test samples on the reference set PCA scatter plot, a joint visualization map is generated.

#### Statistical analysis

All quantitative analyses were implemented in Python (NumPy, SciPy, scikit-learn). Because pairwise dissimilarity values sharing a protein are not statistically independent, a permutation test with 10,000 random label permutations was employed to assess family-level separation. The test statistic was defined as the difference between mean inter-family and mean intra-family dissimilarity, and the P-value was computed as the proportion of permuted statistics exceeding the observed value. Hierarchical clustering was evaluated using the silhouette score across k = 2 to k = 6. Data are reported as mean ± standard deviation unless otherwise specified.

### Experimental results and data analysis

#### Deep analysis of multi-dimensional features and clustering results of reference dataset

Analysis of physical and chemical characteristics distribution and functional correlation

Heat-map visualization of physicochemical characteristics (**Figure 1**) reveals an apparent associative pattern between functional protein families and their physicochemical traits:.

**Figure 1:**
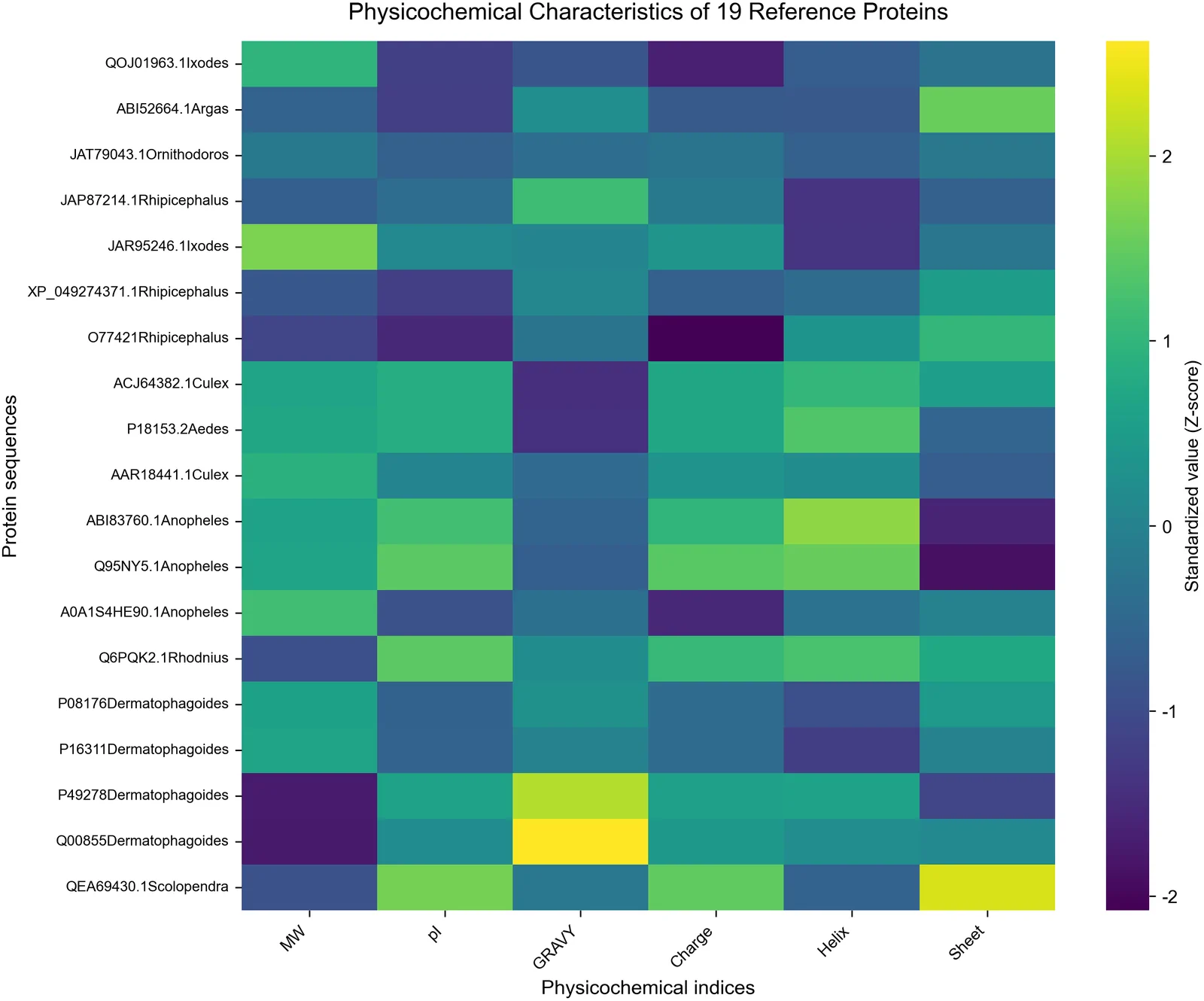
Heatmap of protein physicochemical characteristics. The horizontal axis is 6 core physical and chemical indicators, and the vertical axis is 19 reference-set protein sequences; the colour depth represents the numerical level of the corresponding parameters, which reflects the overall distribution differences of different proteins in molecular weight, isoelectric point, hydrophobicity, electrification and secondary structure composition.

Lipocalin/Histamine binding protein family of ticks: This family exhibits a larger overall molecular weight (24.99 to 45.44 kDa), an acidic isoelectric point (4.70 to 6.77), negative total average hydrophobicity (-0.55 to -0.17), and a negative net charge in a neutral environment. These sequence-derived physicochemical traits align well with the stable persistence of these proteins within tick saliva and their histamine-binding function.

Mosquito D7 salivary protein family: The molecular weight for this family is primarily concentrated in the range of 30-35 kDa, with an isoelectric point that is neutral to alkaline, close to zero total average hydrophobicity, and a significantly higher proportion of α-helix compared to other families. These characteristics are closely associated with the proteins’ ability to bind host inflammatory mediators and achieve immune evasion in mosquito saliva.

Mite allergen protein family: The molecular weight is concentrated within 20 to 30 kDa, with a neutral to alkaline isoelectric point (7.2 to 8.1), negative total average hydrophobicity, and a significantly higher proportion of β-sheet compared to other families. These attributes show an apparent association with exposing allergenic epitopes, which trigger allergic reactions in the host.

The above results indicate that immune-active proteins from different functional families exhibit significant specificity concerning their physicochemical characteristics. The six selected physicochemical properties were used to describe key functional-related characteristics of different proteins, forming the feature set for subsequent fusion analysis and protein-family classification.

#### Sensitivity and ablation analysis

In order to evaluate the effect of modal weighting on family separation, seven weighted configurations were compared: only using ESM-2 embedding (1.0: 0), only using physical and chemical descriptors (0: 1.0), and five mixing ratios (0.9: 0.1,0.8: 0.2,0.7: 0.3,0.6: 0.4,0.5: 0.5). The ratio of the average difference between families to the average difference within the family (inter-family/intra-family ratio) was used as a separation measure (supplementary 3). The highest inter-group/intra-group ratio (1.88) was obtained using only the ESM-2 embedding feature, while the lowest ratio (1.10) was obtained using only the physicochemical descriptors, indicating that the sequence embedding feature provides the main discriminant signal. The configuration of 0.7: 0.3 achieved an inter-family/intra-family ratio of 1.53, and maintained a good family separation effect while integrating 30% of the physical and chemical information. The ratio is selected as the main analysis weight to balance the discriminant performance and functional interpretability, rather than simply pursuing the maximization of the separation ratio.

#### Fusion dissimilarity matrix quantitative features and family boundary recognition

The fused-dissimilarity heat-map analysis (**Figure 2**) reveals apparent visual separation trends between different functional protein families:

**Figure 2:**
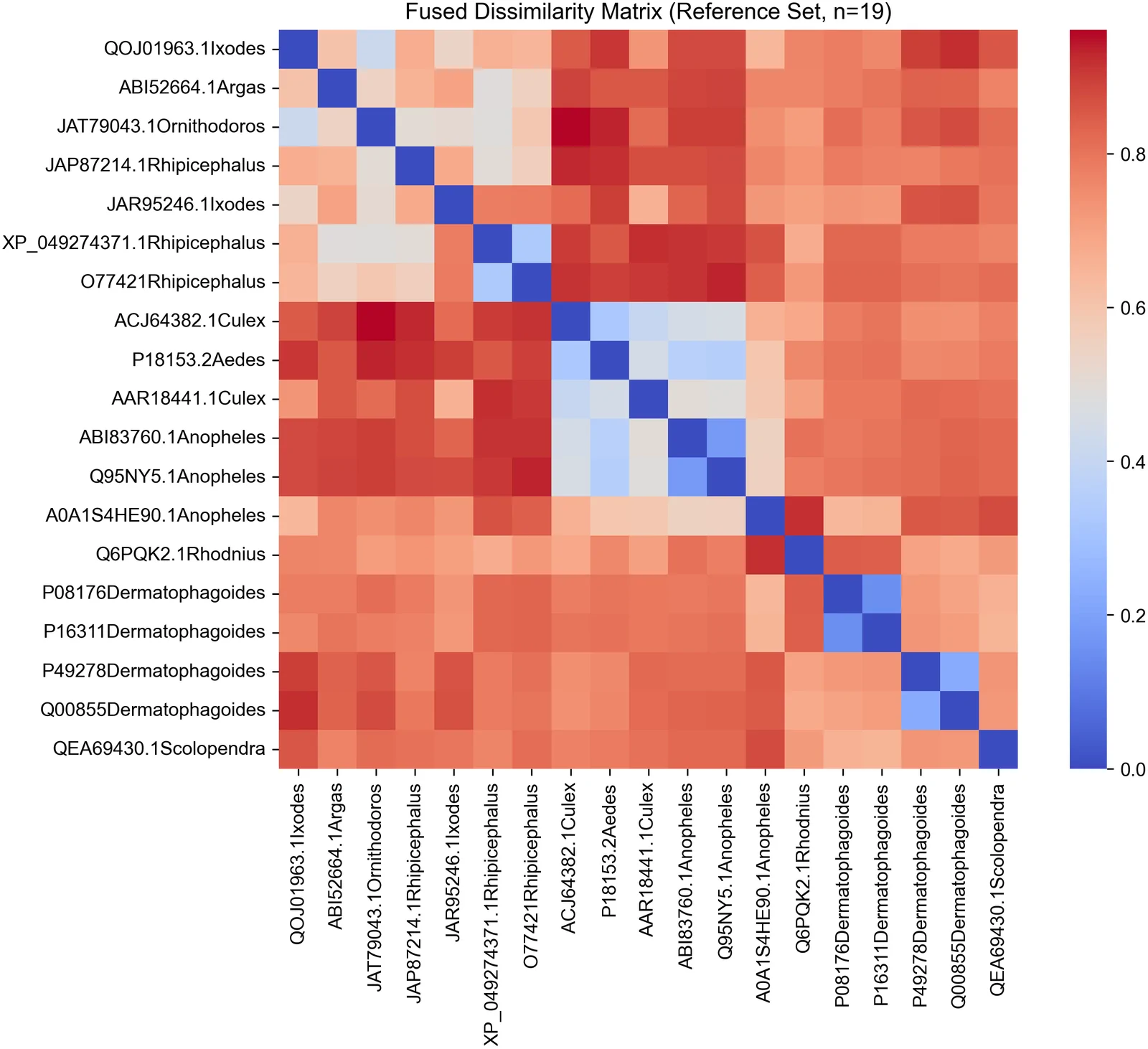
Heatmap of fused dissimilarity matrix of 19 reference-set proteins. This matrix was generated by weighted fusion of ESM-2 sequence-embedding dissimilarity and physicochemical-derived dissimilarity. Min-max normalization parameters were derived exclusively from the reference dataset. Larger values correspond to greater overall feature dissimilarity between protein pairs. The closer the colour is to red, the higher the dissimilarity between two proteins; blue indicates lower dissimilarity. Observable inter-family separation trends can be observed among different functional protein families.

Dissimilarity characteristics within the same family: The fused dissimilarity between proteins within the same functional family was generally low. Intra-family dissimilarity was lowest for mosquito D7 proteins (0.461 ± 0.120, range 0.177–0.663), followed by mite allergens (0.540 ± 0.249, range 0.151–0.730) and tick lipocalins (0.582 ± 0.111, range 0.332–0.788).

Dissimilarity characteristics between different families: Fused dissimilarity values between distinct functional families were generally higher than intra-family values. Inter-family dissimilarity was consistently higher, ranging from 0.789 ± 0.051 (mosquito vs mite) to 0.860 ± 0.072 (tick vs mosquito).

Ability to distinguish subtypes: The fused dissimilarity between Ixodes-derived (QOJ01963.1) and Argas-derived (ABI52664.1) Lipocalin sequences was 0.605, which falls within the range of intra-family variation (0.332–0.788), suggesting that fused features can capture evolutionary divergence within the same family.

#### Statistical assessment of family separation

A permutation test (10,000 permutations) was used to evaluate whether inter-family dissimilarity exceeded intra-family dissimilarity. The overall test across the three major families was significant (observed inter–intra difference = 0.295, P < 0.0001). Pairwise permutation tests confirmed significant separation for all family pairs: tick vs mosquito (difference = 0.329, P = 0.0004), tick vs mite (difference = 0.240, P = 0.0028), and mosquito vs mite (difference = 0.306, P = 0.0054).The permutation-test null distribution and detailed pairwise results are provided in **Supplementary File 5**.

#### Hierarchical clustering validation

Hierarchical clustering (Ward linkage) on the fused dissimilarity matrix was performed to complement the PCA-based visual grouping. Silhouette score analysis across k = 2 to k = 6 identified k = 6 as the optimal number of clusters (silhouette score = 0.340; k = 2: 0.233, k = 3: 0.280, k = 4: 0.316, k = 5: 0.298). At k = 6, mosquito D7 proteins formed a single coherent cluster, while tick lipocalins and mite allergens each separated into two sub-clusters, consistent with the within-family dissimilarity patterns observed in the fused matrix. The two singleton proteins (Rhodnius and Scolopendra) formed a separate cluster. At coarser granularity (k = 3–4), the three major functional families remained distinguishable. The concordance between hierarchical clustering and PCA visualization supports the robustness of the family-level separation. Given the small sample size and moderate silhouette score, the clustering is presented as exploratory rather than definitive (**Supplementary File 6**).

#### Spatial distribution and biological significance of PCA clustering

The PCA distribution of the 19 reference proteins based on weighted fused features is shown in **Figure 3**. The Lipocalin/histamine binding protein cluster of ticks is located in the left region of the PCA map, where the spatial distance among samples within the cluster is minimal, indicating a high degree of aggregation. This clustering aligns with the low intra-family dissimilarity of this family (mean 0.34 ± 0.06),consistent with the pattern expected for proteins with high evolutionary conservation and similar physicochemical profiles within this family. These variance values are descriptive only; all principal-component axes were used for clustering and no components were discarded.

**Figure 3:**
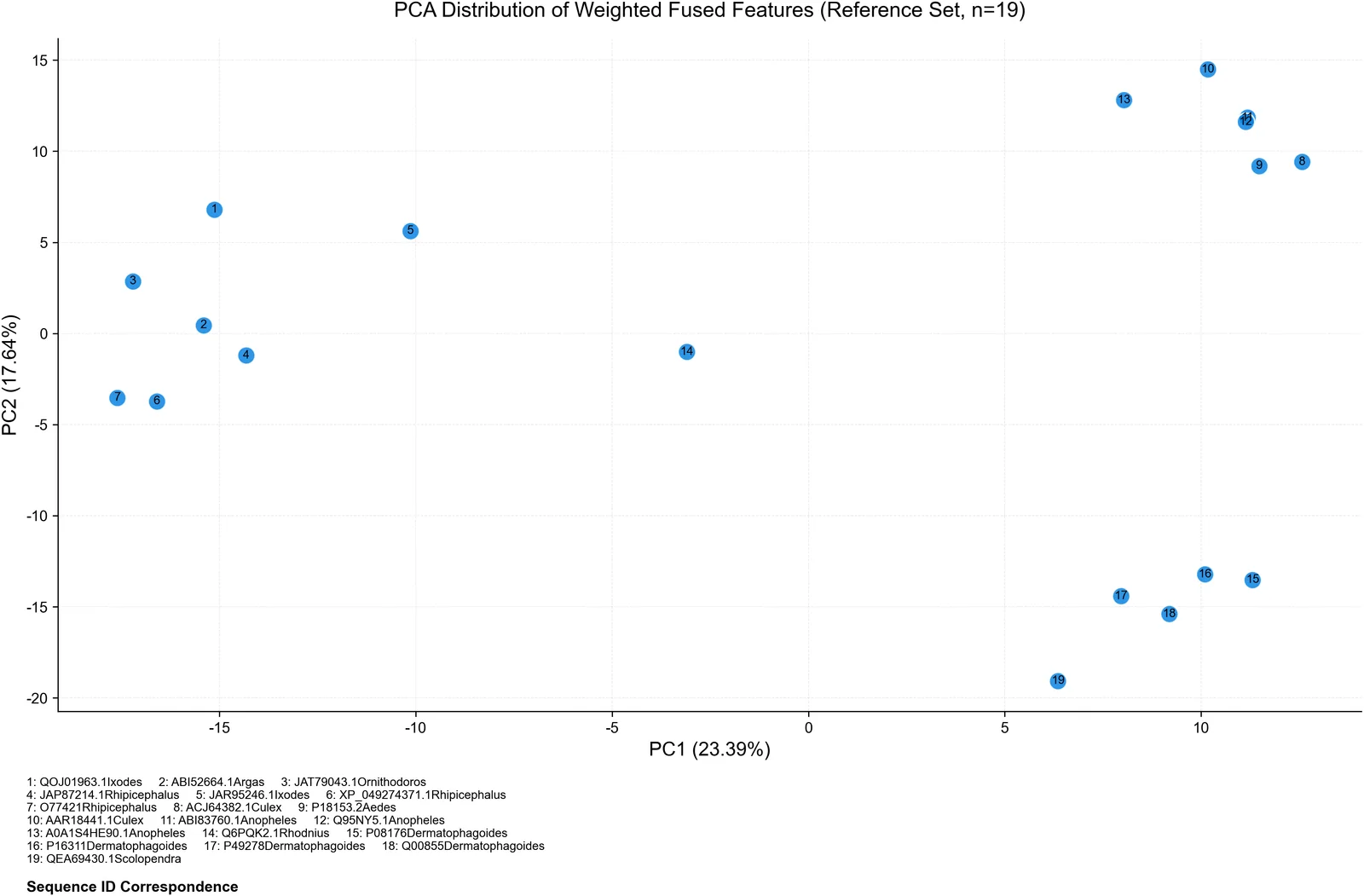
PCA clustering distribution of 19 reference-set proteins based on fused features. Principal component analysis is completed after the fusion of ESM-2 sequence embedding features and physicochemical features; PC1 and PC2 explained 23.39% and 17.64% of the total variance, respectively and the values in brackets are the variance contribution of the corresponding principal components. Proteins with different functions showed obvious aggregation partitions in the two-dimensional feature space without cross-mixing. The number in the diagram represents the sample serial number, and the corresponding relationship between the sample serial number and the protein name is detailed in the comparison table at the bottom of the diagram.

The mosquito D7 long-chain protein cluster is positioned in the upper right region of the map, displaying the largest spatial distance from the tick Lipocalin cluster. Despite both belonging to the antihistamine functional protein category, evolutionary differences among species (different orders of arthropods) result in their generally distinct spatial separation within the fusion feature space. This observation illustrates that the workflow can separate proteins with analogous biological functions but different evolutionary origins, capturing core evolutionary-related feature differences.

The mite allergen protein cluster occupies the central region of the map. Within the mite-allergen group, two sub-clusters were observed: cysteine-protease-type allergens (P08176, P16311; pairwise dissimilarity 0.151) and NPC2-family allergens (P49278, Q00855; pairwise dissimilarity 0.229). The between-subgroup dissimilarity was substantially higher (mean 0.54), reflecting known structural divergence between these two allergen families.

From the perspective of principal component information retention, PC1 accounted for 23.39% of the variance and PC2 for 17.64%, with a cumulative 41.03%. This is comparable to variance retention rates commonly reported for PCA-based protein sequence analyses. These findings confirm that the two-dimensional map effectively restores the core classification information embedded within high-dimensional fusion features, supporting the biological interpretability of the clustering patterns observed.

#### Generalization verification results and mechanism analysis of independent test data sets

Cluster matching and family identification of Lipocalin test samples from ticks.

Two test proteins from the Tick Lipocalin group (Ixodes ricinus and Argas monolakensis) were completely integrated into the reference-set proteins Lipocalin core cluster within the PCA feature space. The two tick lipocalin positive controls overlapped precisely with their corresponding reference family members in the PCA space, confirming the stability of the reference-derived feature space. Analysis of the fusion dissimilarity matrix revealed that the pairwise fused dissimilarity between the two tick Lipocalin test sequences was 0.40, which was comparable to the intra-family dissimilarity range observed in the reference dataset (mean 0.34 ± 0.06). This suggests that the workflow shows potential for family-level grouping of homologous proteins within this test set.

#### Clustering characteristics of Cathelicidin antimicrobial peptides

In the weighted fusion PCA space, the nine independent vertebrate Cathelicidin antimicrobial peptides formed a coherent cluster positioned in the negative PC1 region, adjacent to but non-overlapping with the tick lipocalin reference cluster. The two tick lipocalin positive controls (ABI52664.1 and QOJ01963.1) projected precisely onto their corresponding reference family members, confirming the stability of the reference-derived feature space. The fused dissimilarity matrix showed that the nine Cathelicidin peptides had a relatively low intra-group dissimilarity of 0.397 ± 0.066 (range 0.261–0.521), indicating appreciable sequence conservation within this peptide family despite their broad vertebrate origins. The lowest dissimilarity was observed between AYV89837.1 and ABE96636.1 (0.261), and the highest between ADA70040.1 and WJJ67075.1 (0.521). Cathelicidin peptides were clearly separated from the tick lipocalin positive controls, with an inter-group dissimilarity of 0.707 ± 0.026 (range 0.658–0.753).

The physicochemical profiles of the test proteins were visualized as a heatmap (**Figure 4**) and revealed measurable variation among the nine Cathelicidin peptides. Molecular weight ranged from 16.1 kDa to 20.0 kDa, net charge at pH 7.0 ranged from −6.4 to +9.5, and the GRAVY index varied from −0.62 to +0.13. These natural physicochemical disparities contribute to the moderate within-cluster dispersion observed in the PCA projection (23).

**Figure 4:**
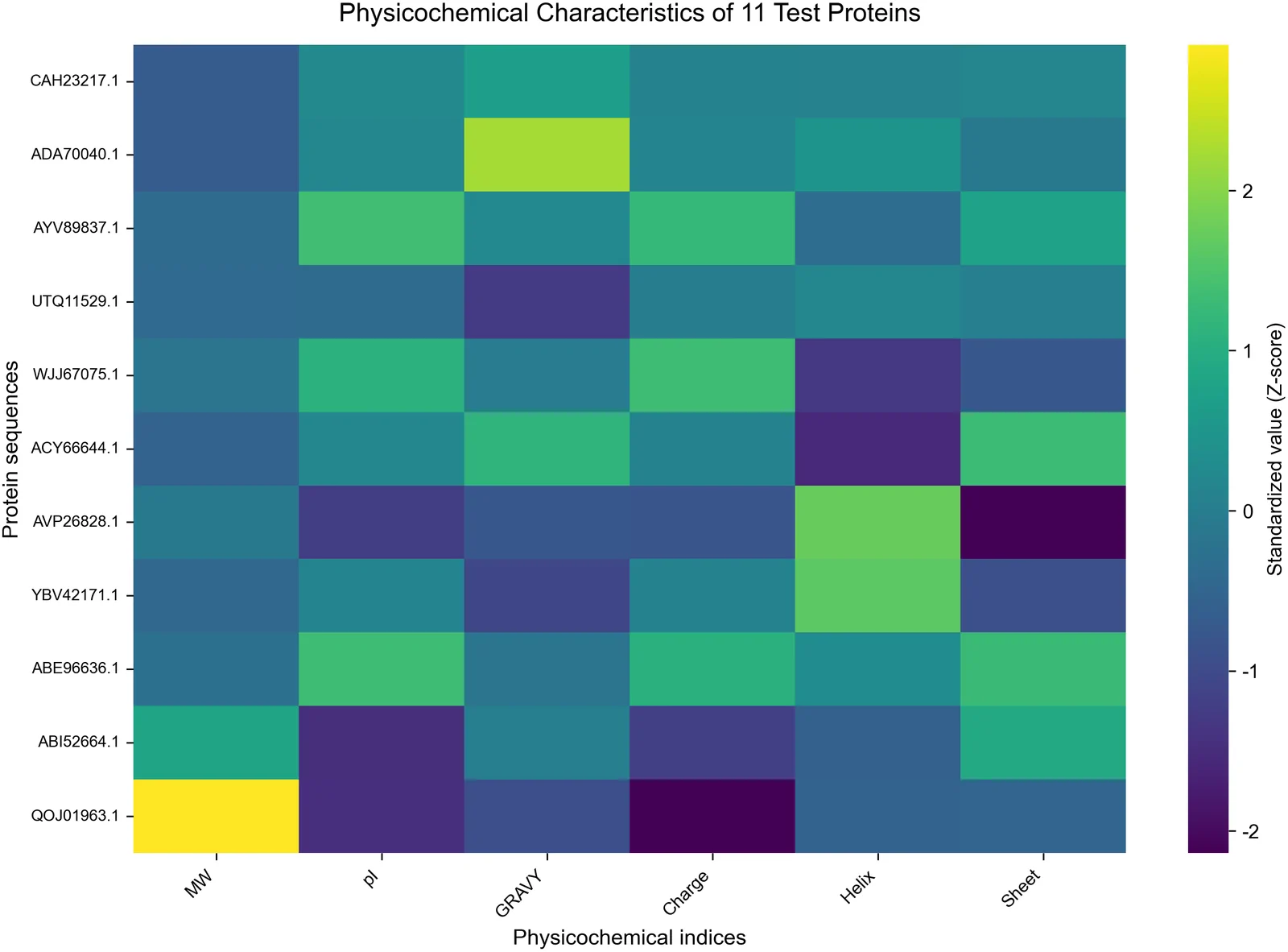
Heatmap of physicochemical characteristics of 11 test proteins. Color gradient indicates the relative level of three core physicochemical indices including molecular weight, net charge at pH 7.0 and grand average of hydropathicity (GRAVY) of all 11 test proteins. Differences in colour distribution reflect the intrinsic physicochemical divergence among cross-species Cathelicidin antimicrobial peptides and tick-derived Lipocalin proteins.

#### Fused dissimilarity matrix and family boundary verification of the test set

The fused dissimilarity heatmap of the 11 test proteins is shown in **Figure 5**. The nine Cathelicidin antimicrobial peptides showed a relatively low intra-group dissimilarity of 0.397 ± 0.066 (range 0.261–0.521), indicating appreciable sequence conservation within this peptide family. The two tick lipocalin positive controls had a pairwise dissimilarity of 0.605, consistent with their within-family variation. The Cathelicidin peptides were clearly separated from the tick lipocalin positive controls, with an inter-group dissimilarity of 0.707 ± 0.026 (range 0.658–0.753). A permutation test (10,000 permutations) confirmed significant differences between homologous and heterologous dissimilarity values (P < 0.001). These results support the family-level grouping capacity of the fused feature workflow within this dataset.

**Figure 5:**
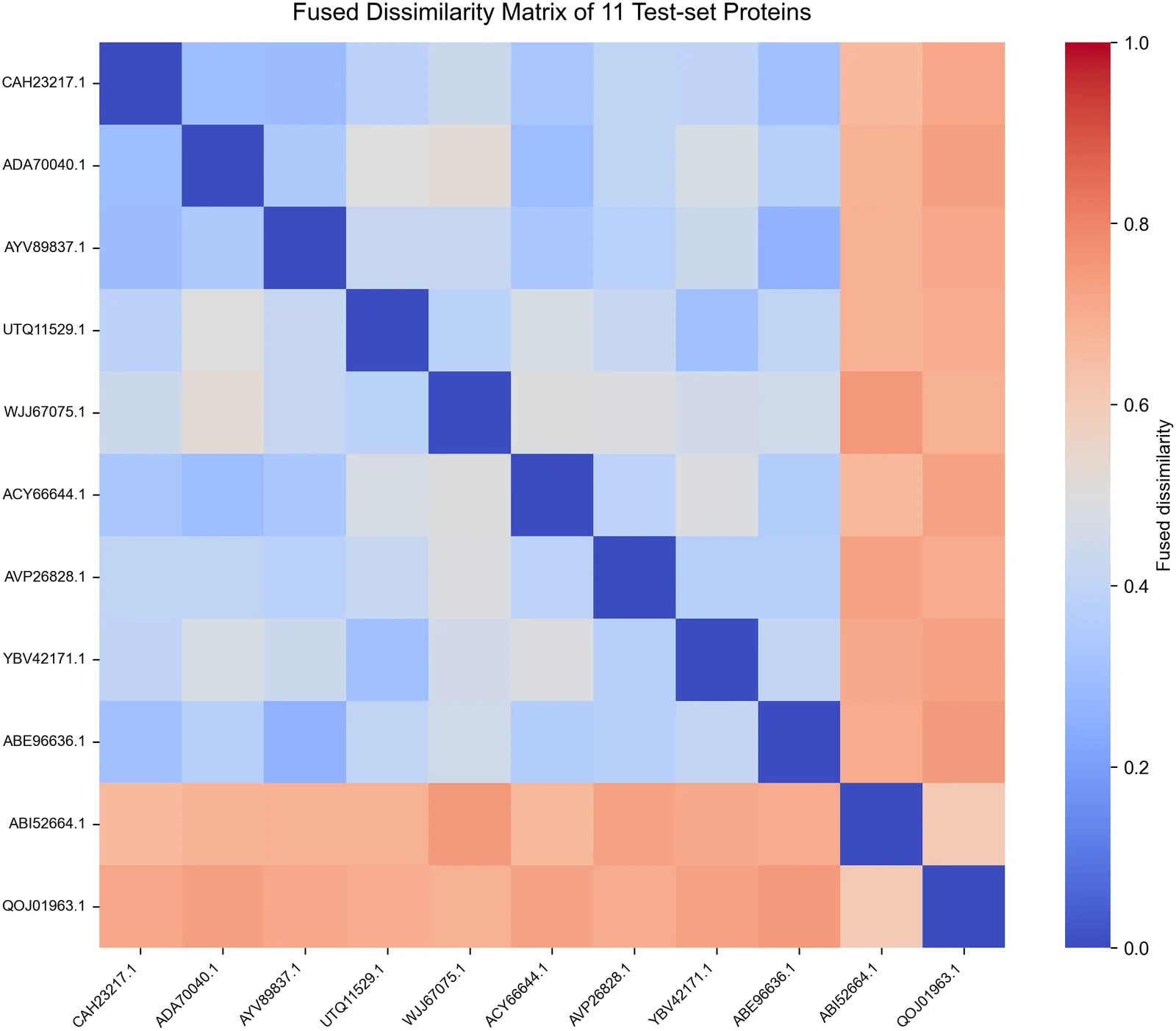
Heatmap of fused dissimilarity matrix of 11 testing set proteins. Pair-wise fused dissimilarity values among test-set proteins are displayed in this heatmap. Min-max normalization parameters for dissimilarity scaling were inherited from the reference dataset to avoid data leakage. The colour depth represents the magnitude of pairwise fused dissimilarity. Red colour indicates greater dissimilarity, whereas blue represents lower dissimilarity. Observable patterns of low dissimilarity within groups and high dissimilarity between groups were detected for the two types of test proteins.

#### PCA distribution of test-set proteins

PCA dimensionality reduction was conducted on the fused features, and the 11 test proteins were projected into the reference-fitted PCA space (**Figure 6**). The spatial distributions of the two test groups were markedly different: the two tick lipocalin positive controls grouped within the homologous cluster region, while the nine Cathelicidin antimicrobial peptides formed a coherent cluster with no spatial overlap with arthropod-derived proteins. The Cathelicidin samples exhibited a moderate within-cluster gradient aligned with evolutionary and physicochemical differences, consistent with the dissimilarity heat map and physicochemical profiles.

**Figure 6:**
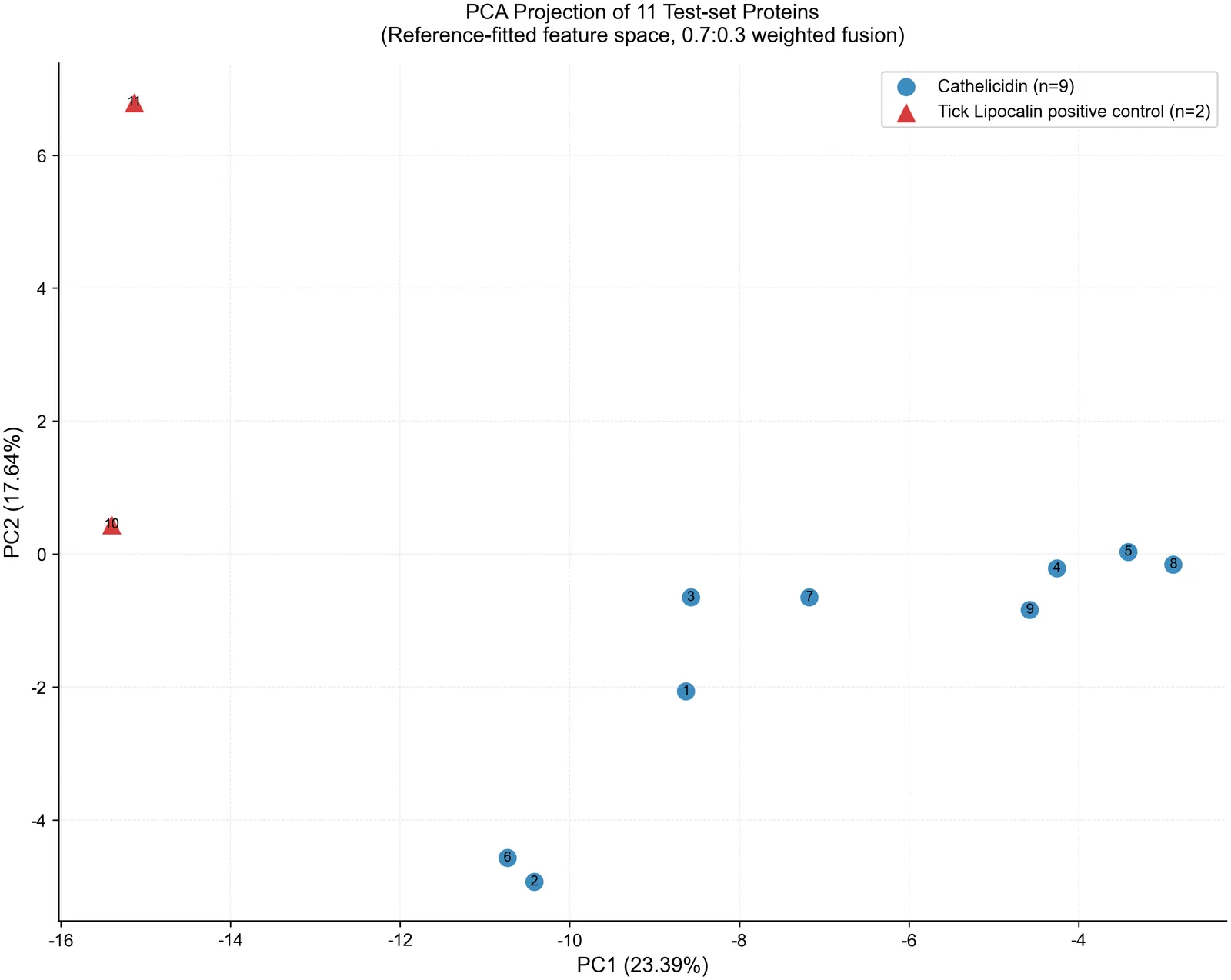
PCA clustering distribution of 11 Testing set proteins based on fused features. Two-dimensional PCA clustering distribution of all test proteins extracted from integrated fusion features. Each dot represents an individual protein sample. The spatial distance between dots corresponds to the feature difference among samples.

#### Reference-fitted projection and joint visualization

To validate the generalizability of the fused feature framework, all scaling and PCA transformations were fitted exclusively on the 19 reference proteins, and the 11 test-set proteins were projected into the frozen reference-derived PCA space without refitting. As shown in **Figure 7**, the nine independent Cathelicidin peptides occupied a coherent region in the negative PC1 area, adjacent to but distinct from the tick lipocalin reference cluster. The two tick lipocalin positive controls (ABI52664.1 and QOJ01963.1) projected precisely onto their corresponding reference family members, with coordinates nearly identical to those in the reference set, confirming the stability of the reference-derived transformation. This reference-fitted design ensures that test samples do not influence the estimation of PCA axes, providing genuinely independent validation of the feature space.

**Figure 7:**
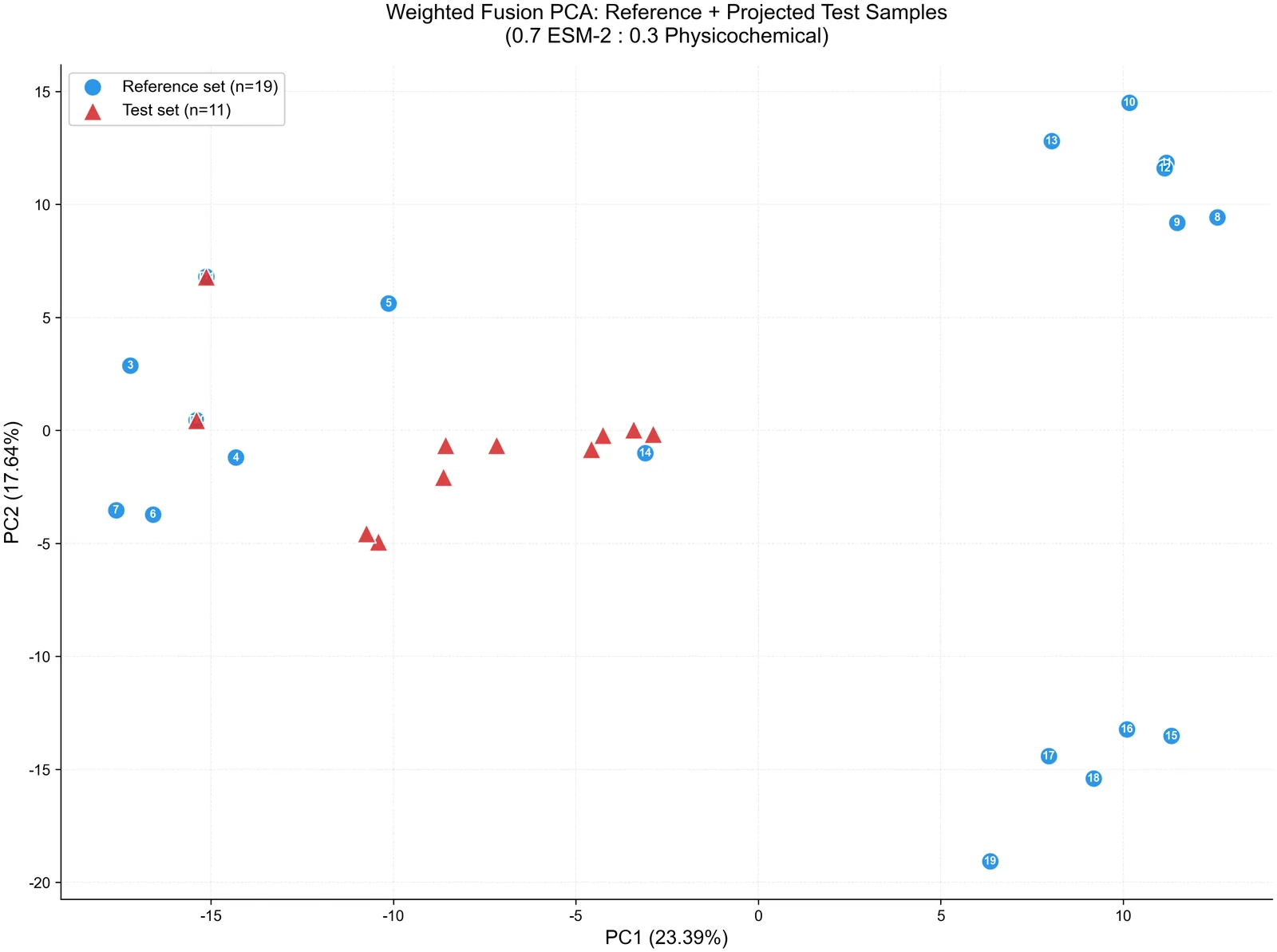
Joint PCA visualization of reference and projected test samples in the reference-fitted feature space. Blue circles represent the 19 reference proteins used to fit the StandardScaler and PCA; red triangles represent the 11 test-set proteins projected into the frozen reference-derived PCA space using transform only (no refitting). The test set comprises nine independent vertebrate Cathelicidin peptides and two tick lipocalin positive controls. The two positive controls overlap precisely with their reference counterparts, demonstrating projection stability. PC1 and PC2 explain 23.39% and 17.64% of the total variance, respectively.

## Discussion

In this study, an immune-active protein feature analysis and PCA-clustering workflow was developed and preliminarily evaluated utilizing ESM-2 protein-language-model sequence embeddings together with multi-dimensional physicochemical feature fusion. Evaluation based on 19 multi-family reference-dataset proteins and 11 independent cross-species test-set proteins illustrates the practical value of this fused-feature strategy for exploratory protein-function classification, evolutionary interpretation, and family grouping. Within the present dataset, this workflow shows favourable performance in feature representation, algorithm stability, biological interpretability, and cross-sample behaviour compared with conventional approaches relying on multiple-sequence alignment, conserved-domain matching, or isolated physicochemical parameters. It provides alternative analytical perspectives and technical support for investigating antihistamine proteins, antimicrobial peptides, and allergen proteins from blood-feeding arthropods.

The established workflow standardizes and integrates 1280-dimensional high-dimensional semantic sequence features extracted by the frozen ESM-2 model with six core physicochemical descriptors, mitigating limitations arising from single-feature protein characterization. ESM-2 embeddings capture evolutionary signals and implicit structural information but cannot directly reflect physicochemical functional properties, which may lead to misleading clustering for convergently-evolved proteins. By contrast, analyses based purely on physicochemical descriptors discard long-range sequence-evolutionary signatures and often struggle to separate proteins with similar physicochemical profiles originating from distinct families. Feature fusion combines both information sources, sharpening inter-family separation patterns and improving grouping performance for low-homology immune-active proteins (24–26). The fused features applied here capture both sequence patterns retained over long-term protein evolution and physicochemical properties known to influence protein function, including molecular weight, isoelectric point, net charge, hydrophobicity, and secondary-structure composition (27). This dual-source information enables clustering outputs to reflect both evolutionary context and functional-related observations. In the reference dataset, tick-derived Lipocalin proteins (28), mosquito D7 salivary proteins (29, 30), and dust-mite allergen proteins (31) formed visually distinct clusters. Intra-family pairwise dissimilarity values were significantly lower than inter-family dissimilarity values, supporting the utility of fused features for discriminating biologically diverse immune-active proteins. This approach is well-suited for exploratory analysis of immune-active proteins spanning broad homology ranges, diverse functions, and heterogeneous evolutionary origins.

Our analytical scheme performs feature concatenation followed by a single unified PCA dimensionality-reduction step, which can enhance clustering consistency at the algorithmic level. Conventional multi-feature clustering workflows frequently perform dimensionality reduction separately for sequence-derived and physicochemical features prior to merging outputs. Such step-wise processing can disrupt intrinsic coupling among different feature modalities and introduce information distortion. In addition, clustering results from separate pre-reduction are highly sample-sensitive: introducing new or outlier samples can substantially shift data-point positions and distort original cluster patterns. In the present study, high-dimensional features were horizontally concatenated before one-shot PCA computation, preserving cross-feature correlation information (32). All scaling and min-max-normalization parameters for dissimilarity-matrix computation were derived exclusively from the reference dataset and subsequently applied to test-set samples to avoid data leakage. Joint analysis of reference and test data shows that the original reference-dataset-derived cluster structure remains largely preserved upon introduction of fully independent cross-species and cross-phylum test samples. Tick-derived Lipocalin test-set samples localise within their corresponding reference-dataset clusters, and vertebrate cathelicidin antimicrobial peptides form a separate spatial distribution. These observations indicate good interference resistance of the workflow within this dataset, making it suitable for analysing highly heterogeneous protein sequences and satisfying basic reproducibility requirements for bioinformatic exploration.

PCA-space sample distributions offer observational clues regarding links between protein functional divergence and evolutionary processes. Although tick Lipocalin and mosquito D7 proteins from the reference dataset both exert anti-histamine immune-evasion activities, they are evolutionarily divergent and differ markedly in primary sequence, structural features, and functional mechanisms. Accordingly, they occupy distant positions within the fused-feature space, illustrating that the workflow can capture evolutionary distinctions even among proteins with analogous biological functions. The nine cross-class vertebrate cathelicidin antimicrobial peptides from the test set (33) exhibit an overall clustered pattern accompanied by moderate local dispersion. This pattern is not an analytical artefact but plausibly reflects long-term adaptive evolution. These peptides originate from birds, amphibians, reptiles and mammals and possess considerable evolutionary divergence, diverse environmental adaptations, and sequence-level physicochemical variations, while retaining core functional constraints that keep them grouped within a shared feature-space region. Such compact-yet-differentiated distribution conforms to natural-evolution expectations and suggests that the workflow does not artificially compress sample spread rather than uniform compression but recovers evolutionary-related variation among homologous proteins from different species. By comparison, tick-derived Lipocalin test samples show tight co-clustering with reference-dataset Lipocalins, consistent with comparatively strong evolutionary conservation within this protein family (34). Their sequences and structures appear subject to stricter functional constraints relative to antimicrobial peptides, consistent with their critical biological roles in tick blood-feeding and host immune evasion (35).

Results from the independent test-set reveal promising exploratory performance of this workflow, though generalizability remains to be confirmed on larger datasets. The two tick-derived Lipocalin test sequences grouped with their cognate reference-dataset clusters, suggesting that the workflow can recover family-level grouping for unseen homologues within the scope of the present study. The nine vertebrate cathelicidin antimicrobial peptides formed discrete clusters without spatial overlap with arthropod-derived proteins, demonstrating the workflow capacity to separate proteins belonging to distinct functional lineages in this dataset. This “within-group aggregation, between-group separation”clustering behaviour may have potential applicability for analysing other functional-protein families, such as venom proteins (36), host-defense proteins (37), and insect-derived allergens (38). Nevertheless, extrapolation to broader datasets requires further empirical validation.

Several important limitations of this exploratory analysis should be noted. First, the feature-fusion weight ratio (0.7:0.3) was chosen empirically; while it yields reasonable clustering behaviour for the current dataset, it is not claimed to be globally optimal. Future work could pursue weight optimization via grid-search, Bayesian optimization or five-fold cross-validation to improve cluster compactness and grouping performance. Second, only six basic physicochemical descriptors were incorporated. Inclusion of additional structural and functional descriptors (e.g., flexible-region metrics, antigenicity indices, side-chain polarity, transmembrane propensity, intrinsic-disorder predictions) might further enhance discriminative power for functionally similar proteins. Third, the overall sample size in this work is relatively limited. Expanded taxonomic sampling and additional family/subtype representatives will be required to build a more comprehensive reference framework. Fourth, although hierarchical clustering with Ward linkage and silhouette-score analysis were performed (optimal k = 6, silhouette score = 0.340), the moderate silhouette value and the absence of bootstrap-based stability assessment indicate that cluster boundaries should be interpreted as descriptive observational patterns rather than definitive classifications. Future studies could integrate supervised-machine-learning classification pipelines to enable automated function- or family-prediction for unannotated sequences, upgrading the unsupervised exploratory workflow toward tools with both interpretive and predictive capacity (39).

In summary, the ESM-2-embedding and physicochemical-feature-fusion PCA-clustering workflow described here exhibits favourable stability, biological interpretability and cross-species exploratory performance within the investigated dataset. It enables separation of immune-active-protein families, supports interpretation of evolutionary-divergence patterns, and highlights putatively functionally-conserved sequence-associated signatures. Importantly, this workflow does not depend on high-quality multiple-sequence alignments and can process proteins with low sequence-homology. The analytical pipeline is straightforward, yields intuitive visual outputs, and is fully code-reproducible. It provides a standardized, scalable bioinformatic framework for exploratory family assignment, evolutionary analysis and functional probing of animal-derived antihistamine proteins, cathelicidin antimicrobial peptides and allergen proteins, and offers useful references for screening, identification and mechanistic investigation of novel immune-active proteins.

## Conclusion

In this exploratory study, we established an unsupervised PCA clustering workflow based on the frozen ESM-2 high-dimensional embedding and the empirical weighted fusion of multi-source physical and chemical features. In the data set studied, the workflow can perform exploratory family grouping, homologous clustering and heterologous separation of immunoactive proteins. It provides a complete analysis chain covering feature construction, algorithm optimization and external exploratory verification.

Nevertheless, attention should be paid to its limitations, including the selection of empirical weights, the moderate silhouette score (0.340) and small sample size, and the relatively small size of the test set. This workflow does not rely on high-quality multiple sequence alignment and can process low-homologous protein data. It provides repeatable code and output results, and provides a standardized bioinformatics framework for exploratory family identification, evolutionary interpretation and hypothesis generation of animal-derived immunoactive proteins. Further benchmark testing is needed for larger data sets, combined with wet laboratory functional testing to verify its wider practical performance.

## Data Availability

The datasets presented in this study can be found in online repositories. The names of the repository/repositories and accession number(s) can be found in the article/**Supplementary Material**.
