# Supplementary material for: Characterization and clustering of immunocompetent proteins based on ESM-2 sequence embedding and physicochemical feature fusion
Source: Front Immunol. 2026 Sep 30;17:1918918. doi: 10.3389/fimmu.2026.1918918 (PMC13661372; doi:10.3389/fimmu.2026.1918918)
Supplement: Supplementary file 1 [file DataSheet1.zip › Supplementary files/The sample charts and Python code are provided in Supplementary File 1/Table.docx]

Table.1 Antihistamine protein sequence list of 19 training sets

| Group ( species example ) | Core antihistamine protein / peptide | Reference sequence ID ( NCBI ) |
| --- | --- | --- |
| Ixodes ricinus | lipocalin | QOJ01963.1 |
| Argas monolakensis | lipocalin | ABI52664.1 |
| Ornithodoros brasiliensis | lipocalin | JAT79043.1 |
| Rhipicephalus appendiculatus | lipocalin | JAP87214 |
| Ixodes ricinus | Histamine-binding protein | JAR95246 |
| Rhipicephalus sanguineus | histamine-binding protein 2-like | XP_049274371 |
| Rhipicephalus appendiculatus | Histamine-binding protein | O77421 |
| Aedes aegypti | Long form salivary protein D7L1 | P18153.2 |
| Culex tarsalis | long form D7 salivary protein | ACJ64382.1 |
| Culex quinquefasciatus | long form D7 salivary protein | AAR18441.1 |
| Anopheles funestus | long form D7 salivary protein | ABI83760.1 |
| Anopheles stephensi | long form D7 salivary protein | Q95NY5.1 |
| Anopheles gambiae | Long form salivary protein D7L | A0A1S4HE90.1 |
| Rhodnius prolixus | Nitrophorin-7 | Q6PQK2.1 |
| Dermatophagoides pteronyssinus | Der p 1 | P08176 |
| Dermatophagoides farinae | Der f 1 | [P16311](https://www.uniprot.org/uniprot/P16311) |
| Dermatophagoides pteronyssinus | Der p 2 | [P49278](https://www.uniprot.org/uniprot/P49278) |
| Dermatophagoides farinae | Der f 2 | [Q00855](https://www.uniprot.org/uniprot/Q00855) |
| Scolopendra subspinipes | Sco m 5 | QEA69430.1 |

Table.2 Protein information table of 11 training sets

| Group ( species example ) | Core antihistamine protein / peptide | Reference sequence ID ( NCBI ) |
| --- | --- | --- |
| Coturnix coturnix | cathelicidin | ADA70040 |
| Phasianus colchicus | cathelicidin | ACY66644 |
| Bubalus bubalis | cathelicidin | CAH23217 |
| Quasipaa spinosa | cathelicidin | YBV42171 |
| Leptobrachium liui | cathelicidin | WJJ67075 |
| Hoplobatrachus rugulosus | cathelicidin | UTQ11529 |
| Crocodylus siamensis | cathelicidin | AYV89837 |
| Python bivittatus | cathelicidin | AVP26828 |
| Saguinus oedipus | cathelicidin | ABE96636 |
| Ixodes ricinus | lipocalin | QOJ01963.1 |
| Argas monolakensis | lipocalin | ABI52664.1 |
