# Supplementary material for: Characterization and clustering of immunocompetent proteins based on ESM-2 sequence embedding and physicochemical feature fusion
Source: Front Immunol. 2026 Sep 30;17:1918918. doi: 10.3389/fimmu.2026.1918918 (PMC13661372; doi:10.3389/fimmu.2026.1918918)
Supplement: Supplementary file 1 [file DataSheet1.zip › Supplementary files/The sample charts and Python code are provided in Supplementary File 3/Sensitivity and ablation analysis/code.docx]

import torch

import numpy as np

import pandas as pd

from Bio import SeqIO

from Bio.SeqUtils import ProtParam

from scipy.spatial.distance import cosine, euclidean

from sklearn.preprocessing import StandardScaler

import matplotlib.pyplot as plt

import seaborn as sns

import os

import warnings

warnings.filterwarnings('ignore')

\# ===================== 路径设置 =====================

fasta_path = r"C:\Users\刘星星\Desktop\蜱抗组胺蛋白序列及表格\文章内容\返修回稿 2026.9.1\Supplementary files\The sample charts and Python code are provided in Supplementary File 3\合并后的序列-19.fasta"

save_dir = r"C:\Users\刘星星\Desktop\蜱抗组胺蛋白序列及表格\文章内容\返修回稿 2026.9.1\Supplementary files\The sample charts and Python code are provided in Supplementary File 3"

os.makedirs(save_dir, exist_ok=True)

\# ===================== 读取序列 =====================

def load_sequences(fasta_file):

records = []

for rec in SeqIO.parse(fasta_file, "fasta"):

seq = str(rec.seq).replace("*", "").upper()

records.append((rec.id, seq))

return records

sequences = load_sequences(fasta_path)

pids = [p for p, s in sequences]

n = len(pids)

print(f"✅ 加载参考集序列 {n} 条")

print("\n===== 序列索引核对（请据此修改下方家族分组） =====")

for i, pid in enumerate(pids):

print(f" [{i:2d}] {pid}")

\# ===================== 计算6个理化指标 =====================

def compute_physicochemical(seq):

pp = ProtParam.ProteinAnalysis(seq)

mw = pp.molecular_weight()

pI = pp.isoelectric_point()

gravy = pp.gravy()

charge = pp.charge_at_pH(7.0)

helix, sheet, coil = pp.secondary_structure_fraction()

return [mw, pI, gravy, charge, helix, sheet]

phy_features = np.array([compute_physicochemical(seq) for _, seq in sequences])

\# ===================== ESM-2嵌入提取 =====================

import esm

model, alphabet = esm.pretrained.esm2_t33_650M_UR50D()

batch_converter = alphabet.get_batch_converter()

model.eval()

device = "cuda" if torch.cuda.is_available() else "cpu"

model.to(device)

def get_esm_embeddings(seq_data):

batch_labels, batch_strs, batch_tokens = batch_converter(seq_data)

batch_tokens = batch_tokens.to(device)

with torch.no_grad():

results = model(batch_tokens, repr_layers=[33], return_contacts=False)

token_reps = results["representations"][33].cpu().numpy()

embs = []

for i, (_, seq) in enumerate(seq_data):

seq_len = len(seq)

emb = token_reps[i, 1:seq_len + 1].mean(0)

embs.append(emb)

return np.array(embs)

embs = get_esm_embeddings(sequences)

print("\n✅ ESM嵌入提取完成")

\# ===================== 标准化 =====================

phy_scaler = StandardScaler()

phy_norm = phy_scaler.fit_transform(phy_features)

emb_scaler = StandardScaler()

embs_norm = emb_scaler.fit_transform(embs)

\# ===================== 构建基础相异度矩阵 =====================

\# 1. ESM余弦距离

S_emb = np.zeros((n, n))

for i in range(n):

for j in range(n):

S_emb[i, j] = cosine(embs_norm[i], embs_norm[j])

\# 2. 理化相异度

D_phy = np.zeros((n, n))

for i in range(n):

for j in range(n):

d = euclidean(phy_norm[i], phy_norm[j])

D_phy[i, j] = 1.0 - 1.0 / (1.0 + d)

\# min-max归一化

semb_min, semb_max = S_emb.min(), S_emb.max()

dphy_min, dphy_max = D_phy.min(), D_phy.max()

S_emb_norm = (S_emb - semb_min) / (semb_max - semb_min)

D_phy_norm = (D_phy - dphy_min) / (dphy_max - dphy_min)

\# ============================================================

\# 【必须核对】家族分组索引——根据上面打印的序列顺序修改

\# ============================================================

\# 示例：假设前7条=tick lipocalin，接下来6条=mosquito D7，最后4条=mite allergen

\# （19条 = 7 tick + 6 mosquito + 4 mite + 2 other/toxin，toxin不纳入消融计算）

\# 请根据实际打印结果修改！

tick_idx = [0, 1, 2, 3, 4, 5, 6] # tick lipocalin 7条

mosquito_idx = [7, 8, 9, 10, 11, 12] # mosquito D7 6条

mite_idx = [15, 16, 17, 18] # mite allergen 4条（根据实际位置调整）

family_groups = [tick_idx, mosquito_idx, mite_idx]

family_names = ["Tick_Lipocalin", "Mosquito_D7", "Mite_allergen"]

print("\n===== 家族分组确认 =====")

for name, group in zip(family_names, family_groups):

print(f" {name} ({len(group)}条):")

for i in group:

print(f"	[{i}] {pids[i]}")

\# ============================================================

\# 消融实验：7种权重比例

\# ============================================================

print("\n===== 消融实验开始（19条参考集） =====")

weight_configs = [

(1.0, 0.0, "ESM_only (1.0:0)"),

(0.9, 0.1, "0.9:0.1"),

(0.8, 0.2, "0.8:0.2"),

(0.7, 0.3, "0.7:0.3 (primary)"),

(0.6, 0.4, "0.6:0.4"),

(0.5, 0.5, "0.5:0.5"),

(0.0, 1.0, "Phy_only (0:1.0)"),

]

ablation_results = []

for w_emb, w_phy, label in weight_configs:

\# 构建加权融合相异度矩阵

if w_emb == 0:

F_w = D_phy_norm.copy()

elif w_phy == 0:

F_w = S_emb_norm.copy()

else:

F_w = w_emb * S_emb_norm + w_phy * D_phy_norm

\# 组内相异度（每个家族内部两两配对）

intra_vals = []

for group in family_groups:

for i in range(len(group)):

for j in range(i + 1, len(group)):

intra_vals.append(F_w[group[i], group[j]])

intra_mean = np.mean(intra_vals)

intra_std = np.std(intra_vals)

\# 组间相异度（不同家族之间两两配对）

inter_vals = []

for gi in range(len(family_groups)):

for gj in range(gi + 1, len(family_groups)):

for i in family_groups[gi]:

for j in family_groups[gj]:

inter_vals.append(F_w[i, j])

inter_mean = np.mean(inter_vals)

inter_std = np.std(inter_vals)

\# 核心指标：inter/intra比值，越大说明家族分离越好

ratio = inter_mean / intra_mean if intra_mean > 0 else float('inf')

ablation_results.append({

'Configuration': label,

'w_ESM': w_emb,

'w_Physicochemical': w_phy,

'Intra_dissim_mean': round(intra_mean, 4),

'Intra_dissim_std': round(intra_std, 4),

'Inter_dissim_mean': round(inter_mean, 4),

'Inter_dissim_std': round(inter_std, 4),

'Inter_Intra_ratio': round(ratio, 4),

})

print(f" {label:25s} | intra={intra_mean:.4f}±{intra_std:.4f} | "

f"inter={inter_mean:.4f}±{inter_std:.4f} | ratio={ratio:.4f}")

\# ===================== 保存结果表格 =====================

df_ablation = pd.DataFrame(ablation_results)

csv_path = os.path.join(save_dir, "ablation_analysis_results_19ref.csv")

df_ablation.to_csv(csv_path, encoding='utf-8-sig', index=False)

print(f"\n✅ 消融结果已保存: {csv_path}")

\# ===================== 消融对比图 =====================

plt.rcParams['font.sans-serif'] = ['Arial']

plt.rcParams['axes.unicode_minus'] = False

fig, axes = plt.subplots(1, 2, figsize=(16, 6))

\# 左图：inter/intra ratio柱状图

labels = [r['Configuration'] for r in ablation_results]

ratios = [r['Inter_Intra_ratio'] for r in ablation_results]

colors = ['#b2bec3'] * len(labels)

for i, l in enumerate(labels):

if '0.7:0.3' in l:

colors[i] = '#d63031' # 主分析高亮红色

bars = axes[0].bar(labels, ratios, color=colors, edgecolor='white', linewidth=1.2)

axes[0].set_ylabel('Inter-/Intra-family Dissimilarity Ratio', fontsize=12)

axes[0].set_xlabel('Weight Configuration (ESM : Physicochemical)', fontsize=12)

axes[0].set_title('Ablation: Family Separation by Weight Ratio\n(Reference Set, n=19)', fontsize=13, pad=12)

axes[0].tick_params(axis='x', rotation=30)

for bar, val in zip(bars, ratios):

axes[0].text(bar.get_x() + bar.get_width() / 2, bar.get_height() + 0.02,

f'{val:.2f}', ha='center', va='bottom', fontsize=9)

axes[0].grid(axis='y', alpha=0.3, linestyle='--')

axes[0].spines['top'].set_visible(False)

axes[0].spines['right'].set_visible(False)

\# 右图：组内 vs 组间相异度对比

x = np.arange(len(labels))

width = 0.35

intra_means = [r['Intra_dissim_mean'] for r in ablation_results]

inter_means = [r['Inter_dissim_mean'] for r in ablation_results]

axes[1].bar(x - width / 2, intra_means, width, label='Intra-family', color='#74b9ff', edgecolor='white')

axes[1].bar(x + width / 2, inter_means, width, label='Inter-family', color='#fd79a8', edgecolor='white')

axes[1].set_ylabel('Mean Dissimilarity', fontsize=12)

axes[1].set_xlabel('Weight Configuration', fontsize=12)

axes[1].set_title('Intra- vs Inter-family Dissimilarity\n(Reference Set, n=19)', fontsize=13, pad=12)

axes[1].set_xticks(x)

axes[1].set_xticklabels(labels, rotation=30, ha='right')

axes[1].legend(fontsize=10)

axes[1].grid(axis='y', alpha=0.3, linestyle='--')

axes[1].spines['top'].set_visible(False)

axes[1].spines['right'].set_visible(False)

plt.tight_layout()

fig_path = os.path.join(save_dir, "ablation_weight_ratio_comparison_19ref.png")

plt.savefig(fig_path, dpi=300, bbox_inches='tight')

plt.close()

print(f"✅ 消融对比图已保存: {fig_path}")

\# ===================== 每个家族单独的消融明细 =====================

print("\n===== 各家族组内相异度明细（按权重） =====")

detail_rows = []

for w_emb, w_phy, label in weight_configs:

if w_emb == 0:

F_w = D_phy_norm.copy()

elif w_phy == 0:

F_w = S_emb_norm.copy()

else:

F_w = w_emb * S_emb_norm + w_phy * D_phy_norm

row = {'Configuration': label}

for name, group in zip(family_names, family_groups):

vals = []

for i in range(len(group)):

for j in range(i + 1, len(group)):

vals.append(F_w[group[i], group[j]])

row[f'{name}_intra_mean'] = round(np.mean(vals), 4)

detail_rows.append(row)

df_detail = pd.DataFrame(detail_rows)

detail_path = os.path.join(save_dir, "ablation_per_family_detail_19ref.csv")

df_detail.to_csv(detail_path, encoding='utf-8-sig', index=False)

print(df_detail.to_string(index=False))

print(f"\n✅ 各家族明细已保存: {detail_path}")

\# ===================== 输出排名 =====================

ranked = sorted(ablation_results, key=lambda x: -x['Inter_Intra_ratio'])

print("\n===== 消融结果排名（按inter/intra ratio从高到低） =====")

for rank, r in enumerate(ranked, 1):

marker = " ← 主分析" if '0.7:0.3' in r['Configuration'] else ""

print(f" 第{rank}名: {r['Configuration']:25s} ratio={r['Inter_Intra_ratio']:.4f}{marker}")

print("\n🎉 消融实验全部完成！")

print(f"📂 输出目录: {save_dir}")

print("\n生成文件：")

print(" 1. ablation_analysis_results_19ref.csv (7种权重汇总表)")

print(" 2. ablation_per_family_detail_19ref.csv (各家族组内明细)")

print(" 3. ablation_weight_ratio_comparison_19ref.png (双栏对比图)")
