# Supplementary material for: Characterization and clustering of immunocompetent proteins based on ESM-2 sequence embedding and physicochemical feature fusion
Source: Front Immunol. 2026 Sep 30;17:1918918. doi: 10.3389/fimmu.2026.1918918 (PMC13661372; doi:10.3389/fimmu.2026.1918918)
Supplement: Supplementary file 1 [file DataSheet1.zip › Supplementary files/The sample charts and Python code are provided in Supplementary File 3/code.docx]

import torch

import numpy as np

import pandas as pd

from Bio import SeqIO

from Bio.SeqUtils import ProtParam

from scipy.spatial.distance import cosine, euclidean

from sklearn.preprocessing import StandardScaler

from sklearn.decomposition import PCA

import matplotlib.pyplot as plt

import seaborn as sns

import os

import warnings

warnings.filterwarnings('ignore')

\# ===================== 路径 =====================

ref_fasta = r"C:\Users\刘星星\Desktop\蜱抗组胺蛋白序列及表格\文章内容\返修回稿 2026.9.1\Supplementary files\The sample charts and Python code are provided in Supplementary File 3\合并后的序列-19.fasta"

test_fasta = r"C:\Users\刘星星\Desktop\蜱抗组胺蛋白序列及表格\merged-11.fasta" # ← 改为你的11条测试集路径

save_dir = r"C:\Users\刘星星\Desktop\蜱抗组胺蛋白序列及表格\文章内容\返修回稿 2026.9.1\Supplementary files\The sample charts and Python code are provided in Supplementary File 3"

os.makedirs(save_dir, exist_ok=True)

\# ===================== 通用函数 =====================

def load_sequences(fasta_file):

records = []

for rec in SeqIO.parse(fasta_file, "fasta"):

seq = str(rec.seq).replace("*", "").upper()

records.append((rec.id, seq))

return records

def compute_physicochemical(seq):

pp = ProtParam.ProteinAnalysis(seq)

mw = pp.molecular_weight()

pI = pp.isoelectric_point()

gravy = pp.gravy()

charge = pp.charge_at_pH(7.0)

helix, sheet, coil = pp.secondary_structure_fraction()

return [mw, pI, gravy, charge, helix, sheet]

\# ===================== 加载ESM-2 =====================

import esm

model, alphabet = esm.pretrained.esm2_t33_650M_UR50D()

batch_converter = alphabet.get_batch_converter()

model.eval()

device = "cuda" if torch.cuda.is_available() else "cpu"

model.to(device)

def get_esm_embeddings(seq_data):

batch_labels, batch_strs, batch_tokens = batch_converter(seq_data)

batch_tokens = batch_tokens.to(device)

with torch.no_grad():

results = model(batch_tokens, repr_layers=[33], return_contacts=False)

token_reps = results["representations"][33].cpu().numpy()

embs = []

for i, (_, seq) in enumerate(seq_data):

seq_len = len(seq)

emb = token_reps[i, 1:seq_len+1].mean(0)

embs.append(emb)

return np.array(embs)

\# ===================== 1. 参考集（19条） =====================

ref_sequences = load_sequences(ref_fasta)

ref_pids = [p for p, s in ref_sequences]

n_ref = len(ref_pids)

print(f"✅ 参考集: {n_ref} 条")

for i, pid in enumerate(ref_pids):

print(f" [{i:2d}] {pid}")

ref_phy = np.array([compute_physicochemical(seq) for _, seq in ref_sequences])

ref_embs = get_esm_embeddings(ref_sequences)

\# scaler只用参考集fit

phy_scaler = StandardScaler()

ref_phy_norm = phy_scaler.fit_transform(ref_phy)

emb_scaler = StandardScaler()

ref_embs_norm = emb_scaler.fit_transform(ref_embs)

\# ===================== 2. 测试集（11条，只用transform） =====================

test_sequences = load_sequences(test_fasta)

test_pids = [p for p, s in test_sequences]

n_test = len(test_pids)

print(f"\n✅ 测试集: {n_test} 条")

test_phy = np.array([compute_physicochemical(seq) for _, seq in test_sequences])

test_embs = get_esm_embeddings(test_sequences)

test_phy_norm = phy_scaler.transform(test_phy)

test_embs_norm = emb_scaler.transform(test_embs)

\# ===================== 3. 【问题2核心】加权PCA =====================

alpha = 0.7

beta = 0.3

\# 参考集：加权后拼接

ref_weighted = np.hstack([alpha * ref_embs_norm, beta * ref_phy_norm])

print(f"\n✅ 加权PCA输入维度: {ref_weighted.shape} (1280×0.7 + 6×0.3)")

\# PCA只用参考集fit

pca = PCA(n_components=2)

ref_pca = pca.fit_transform(ref_weighted)

\# 测试集：投影到参考集PCA空间

test_weighted = np.hstack([alpha * test_embs_norm, beta * test_phy_norm])

test_pca = pca.transform(test_weighted)

pc1_var = pca.explained_variance_ratio_[0] * 100

pc2_var = pca.explained_variance_ratio_[1] * 100

print(f"✅ PC1 = {pc1_var:.2f}%")

print(f"✅ PC2 = {pc2_var:.2f}%")

print(f"✅ 累计 = {pc1_var+pc2_var:.2f}%")

\# 保存PCA坐标

pca_df = pd.DataFrame({

'Set': ['Reference']*n_ref + ['Test']*n_test,

'Protein_ID': ref_pids + test_pids,

'PC1': list(ref_pca[:,0]) + list(test_pca[:,0]),

'PC2': list(ref_pca[:,1]) + list(test_pca[:,1]),

})

pca_df.to_csv(os.path.join(save_dir, "weighted_pca_coordinates.csv"), encoding='utf-8-sig', index=False)

\# ===================== 4. 图：参考集单独PCA（正文图3） =====================

plt.rcParams['font.sans-serif'] = ['Arial']

plt.rcParams['axes.unicode_minus'] = False

plt.figure(figsize=(13, 9))

plt.scatter(ref_pca[:,0], ref_pca[:,1], s=140, c='#0984e3', alpha=0.85,

edgecolor='white', linewidth=1.0, zorder=3)

for idx, (x, y) in enumerate(ref_pca):

plt.text(x, y, str(idx+1), fontsize=8, ha='center', va='center', color='black')

plt.xlabel(f"PC1 ({pc1_var:.2f}%)", fontsize=13)

plt.ylabel(f"PC2 ({pc2_var:.2f}%)", fontsize=13)

plt.title("PCA Distribution of Weighted Fused Features (Reference Set, n=19)", fontsize=14, pad=15)

plt.grid(alpha=0.25, linestyle='--', linewidth=0.5)

plt.gca().spines['top'].set_visible(False)

plt.gca().spines['right'].set_visible(False)

plt.figtext(0.03, 0.02, "Sequence ID Correspondence", fontsize=10, weight='semibold')

id_text = ""

for idx, name in enumerate(ref_pids):

id_text += f"{idx+1}: {name}	"

if (idx + 1) % 3 == 0:

id_text += "\n"

plt.figtext(0.03, 0.05, id_text, fontsize=8)

plt.tight_layout(rect=[0, 0.14, 1, 0.96])

plt.savefig(os.path.join(save_dir, "Fig3_weighted_PCA_reference_19.png"), dpi=300, bbox_inches='tight')

plt.close()

print("✅ 图3（参考集PCA）已保存")

\# ===================== 5. 图：联合PCA（参考集+测试集投影，解决问题3可视化） =====================

plt.figure(figsize=(12, 9))

plt.scatter(ref_pca[:,0], ref_pca[:,1], s=140, c='#0984e3', alpha=0.85,

edgecolor='white', linewidth=1.0, label='Reference set (n=19)', zorder=3)

plt.scatter(test_pca[:,0], test_pca[:,1], s=160, c='#d63031', alpha=0.9,

marker='^', edgecolor='white', linewidth=1.0, label='Test set (n=11)', zorder=4)

for idx, (x, y) in enumerate(ref_pca):

plt.text(x, y, str(idx+1), fontsize=7.5, ha='center', va='center', color='white', fontweight='bold')

for idx, (x, y) in enumerate(test_pca):

plt.text(x, y, f'T{idx+1}', fontsize=7.5, ha='center', va='center', color='white', fontweight='bold')

plt.xlabel(f"PC1 ({pc1_var:.2f}%)", fontsize=13)

plt.ylabel(f"PC2 ({pc2_var:.2f}%)", fontsize=13)

plt.title("Weighted Fusion PCA: Reference + Projected Test Samples\n(0.7 ESM-2 : 0.3 Physicochemical)", fontsize=14, pad=15)

plt.legend(fontsize=11, loc='best', framealpha=0.9)

plt.grid(alpha=0.25, linestyle='--', linewidth=0.5)

plt.gca().spines['top'].set_visible(False)

plt.gca().spines['right'].set_visible(False)

plt.tight_layout()

plt.savefig(os.path.join(save_dir, "Fig_joint_PCA_reference_test.png"), dpi=300, bbox_inches='tight')

plt.close()

print("✅ 联合PCA图已保存")

\# ===================== 6. 相异度矩阵（供热图） =====================

\# 参考集

S_emb_ref = np.zeros((n_ref, n_ref))

D_phy_ref = np.zeros((n_ref, n_ref))

for i in range(n_ref):

for j in range(n_ref):

S_emb_ref[i,j] = cosine(ref_embs_norm[i], ref_embs_norm[j])

d = euclidean(ref_phy_norm[i], ref_phy_norm[j])

D_phy_ref[i,j] = 1.0 - 1.0/(1.0 + d)

semb_min, semb_max = S_emb_ref.min(), S_emb_ref.max()

dphy_min, dphy_max = D_phy_ref.min(), D_phy_ref.max()

S_emb_ref_norm = (S_emb_ref - semb_min) / (semb_max - semb_min)

D_phy_ref_norm = (D_phy_ref - dphy_min) / (dphy_max - dphy_min)

F_ref = alpha * S_emb_ref_norm + beta * D_phy_ref_norm

df_F_ref = pd.DataFrame(F_ref, index=ref_pids, columns=ref_pids)

df_F_ref.to_csv(os.path.join(save_dir, "fused_dissimilarity_19ref.csv"), encoding='utf-8-sig')

plt.figure(figsize=(10,8))

sns.heatmap(df_F_ref, cmap="coolwarm", square=True)

plt.title("Fused Dissimilarity Matrix (Reference Set, n=19)", fontsize=12)

plt.tight_layout()

plt.savefig(os.path.join(save_dir, "Fig2_fused_dissimilarity_heatmap_19.png"), dpi=300, bbox_inches='tight')

plt.close()

print("✅ 图2（参考集热图）已保存")

\# 测试集（用参考集的min-max参数）

S_emb_test = np.zeros((n_test, n_test))

D_phy_test = np.zeros((n_test, n_test))

for i in range(n_test):

for j in range(n_test):

S_emb_test[i,j] = cosine(test_embs_norm[i], test_embs_norm[j])

d = euclidean(test_phy_norm[i], test_phy_norm[j])

D_phy_test[i,j] = 1.0 - 1.0/(1.0 + d)

S_emb_test_norm = (S_emb_test - semb_min) / (semb_max - semb_min)

D_phy_test_norm = (D_phy_test - dphy_min) / (dphy_max - dphy_min)

F_test = alpha * S_emb_test_norm + beta * D_phy_test_norm

df_F_test = pd.DataFrame(F_test, index=test_pids, columns=test_pids)

df_F_test.to_csv(os.path.join(save_dir, "fused_dissimilarity_11test.csv"), encoding='utf-8-sig')

plt.figure(figsize=(10,8))

sns.heatmap(df_F_test, cmap="coolwarm", square=True)

plt.title("Fused Dissimilarity Matrix (Test Set, n=11)", fontsize=12)

plt.tight_layout()

plt.savefig(os.path.join(save_dir, "Fig5_fused_dissimilarity_heatmap_11test.png"), dpi=300, bbox_inches='tight')

plt.close()

print("✅ 图5（测试集热图）已保存")

print("\n🎉 全部完成！")

print(f"📂 输出目录: {save_dir}")

print(f"\n⚠️ 稿件必须更新的数值：")

print(f"	PC1 = {pc1_var:.2f}%")

print(f"	PC2 = {pc2_var:.2f}%")

print(f"	参考集样本数 = 19（所有21改为19）")
