# Supplementary material for: Characterization and clustering of immunocompetent proteins based on ESM-2 sequence embedding and physicochemical feature fusion
Source: Front Immunol. 2026 Sep 30;17:1918918. doi: 10.3389/fimmu.2026.1918918 (PMC13661372; doi:10.3389/fimmu.2026.1918918)
Supplement: Supplementary file 1 [file DataSheet1.zip › Supplementary files/The sample charts and Python code are provided in Supplementary File 4/code.docx]

import torch

import numpy as np

import pandas as pd

from Bio import SeqIO

from Bio.SeqUtils import ProtParam

from scipy.spatial.distance import cosine, euclidean

from sklearn.preprocessing import StandardScaler

from sklearn.decomposition import PCA

import matplotlib.pyplot as plt

import seaborn as sns

import os

import warnings

warnings.filterwarnings('ignore')

\# ===================== 路径设置 =====================

ref_fasta = r"C:\Users\刘星星\Desktop\蜱抗组胺蛋白序列及表格\文章内容\返修回稿 2026.9.1\Supplementary files\The sample charts and Python code are provided in Supplementary File 3\合并后的序列-19.fasta"

test_fasta = r"C:\Users\刘星星\Desktop\蜱抗组胺蛋白序列及表格\文章内容\返修回稿 2026.9.1\Supplementary files\The sample charts and Python code are provided in Supplementary File 4\merged-11.fasta"

save_dir = r"C:\Users\刘星星\Desktop\蜱抗组胺蛋白序列及表格\文章内容\返修回稿 2026.9.1\Supplementary files\The sample charts and Python code are provided in Supplementary File 4"

os.makedirs(save_dir, exist_ok=True)

\# ===================== 读取序列 =====================

def load_sequences(fasta_file):

records = []

for rec in SeqIO.parse(fasta_file, "fasta"):

seq = str(rec.seq).replace("*", "").upper()

records.append((rec.id, seq))

return records

ref_sequences = load_sequences(ref_fasta)

test_sequences = load_sequences(test_fasta)

ref_pids = [p for p, s in ref_sequences]

test_pids = [p for p, s in test_sequences]

print(f"✅ 参考集 {len(ref_pids)} 条，测试集 {len(test_pids)} 条")

\# ===================== 计算理化特征 =====================

def compute_physicochemical(seq):

pp = ProtParam.ProteinAnalysis(seq)

mw = pp.molecular_weight()

pI = pp.isoelectric_point()

gravy = pp.gravy()

charge = pp.charge_at_pH(7.0)

helix, sheet, coil = pp.secondary_structure_fraction()

return [mw, pI, gravy, charge, helix, sheet]

ref_phy = np.array([compute_physicochemical(seq) for _, seq in ref_sequences])

test_phy = np.array([compute_physicochemical(seq) for _, seq in test_sequences])

\# 保存测试集理化特征表

df_test_phy = pd.DataFrame(test_phy, index=test_pids, columns=["MW","pI","GRAVY","Charge","Helix","Sheet"])

df_test_phy.to_csv(os.path.join(save_dir, "test_physicochemical_features.csv"), encoding='utf-8-sig')

print("✅ 测试集理化特征已保存")

\# ===================== 加载 ESM-2 =====================

import esm

model, alphabet = esm.pretrained.esm2_t33_650M_UR50D()

batch_converter = alphabet.get_batch_converter()

model.eval()

device = "cuda" if torch.cuda.is_available() else "cpu"

model.to(device)

def get_esm_embeddings(seq_data):

batch_labels, batch_strs, batch_tokens = batch_converter(seq_data)

batch_tokens = batch_tokens.to(device)

with torch.no_grad():

results = model(batch_tokens, repr_layers=[33], return_contacts=False)

token_reps = results["representations"][33].cpu().numpy()

embs = []

for i, (_, seq) in enumerate(seq_data):

seq_len = len(seq)

emb = token_reps[i, 1:seq_len+1].mean(0)

embs.append(emb)

return np.array(embs)

ref_embs = get_esm_embeddings(ref_sequences)

test_embs = get_esm_embeddings(test_sequences)

print("✅ ESM-2 嵌入提取完成")

\# ===================== 用参考集 fit scaler，测试集只 transform =====================

phy_scaler = StandardScaler()

ref_phy_norm = phy_scaler.fit_transform(ref_phy)

test_phy_norm = phy_scaler.transform(test_phy) # 关键：只transform，不fit

emb_scaler = StandardScaler()

ref_embs_norm = emb_scaler.fit_transform(ref_embs)

test_embs_norm = emb_scaler.transform(test_embs) # 关键：只transform，不fit

\# ===================== 参考集相异度矩阵（用于获取min-max参数） =====================

n_ref = len(ref_pids)

S_emb_ref = np.zeros((n_ref, n_ref))

D_phy_ref = np.zeros((n_ref, n_ref))

for i in range(n_ref):

for j in range(n_ref):

S_emb_ref[i,j] = cosine(ref_embs_norm[i], ref_embs_norm[j])

d = euclidean(ref_phy_norm[i], ref_phy_norm[j])

s_phy = 1.0 / (1.0 + d)

D_phy_ref[i,j] = 1.0 - s_phy

\# 参考集全局 min/max（用于测试集归一化）

emb_min, emb_max = S_emb_ref.min(), S_emb_ref.max()

phy_min, phy_max = D_phy_ref.min(), D_phy_ref.max()

print(f"✅ 参考集min-max参数：emb[{emb_min:.4f},{emb_max:.4f}], phy[{phy_min:.4f},{phy_max:.4f}]")

\# ===================== 测试集相异度矩阵（用参考集min-max归一化） =====================

n_test = len(test_pids)

S_emb_test = np.zeros((n_test, n_test))

D_phy_test = np.zeros((n_test, n_test))

for i in range(n_test):

for j in range(n_test):

S_emb_test[i,j] = cosine(test_embs_norm[i], test_embs_norm[j])

d = euclidean(test_phy_norm[i], test_phy_norm[j])

s_phy = 1.0 / (1.0 + d)

D_phy_test[i,j] = 1.0 - s_phy

\# 用参考集min-max归一化（关键：不能用测试集自己的min-max）

S_emb_test_norm = (S_emb_test - emb_min) / (emb_max - emb_min)

D_phy_test_norm = (D_phy_test - phy_min) / (phy_max - phy_min)

\# 裁剪到[0,1]

S_emb_test_norm = np.clip(S_emb_test_norm, 0, 1)

D_phy_test_norm = np.clip(D_phy_test_norm, 0, 1)

\# 0.7:0.3 加权融合

alpha = 0.7

F_test = alpha * S_emb_test_norm + (1 - alpha) * D_phy_test_norm

df_F_test = pd.DataFrame(F_test, index=test_pids, columns=test_pids)

df_F_test.to_csv(os.path.join(save_dir, "fused_dissimilarity_11test.csv"), encoding='utf-8-sig')

print("✅ 测试集融合相异度矩阵已保存")

\# ===================== 用参考集 fit PCA，测试集只 transform（0.7:0.3加权） =====================

\# 参考集加权特征矩阵

ref_weighted = np.hstack([0.7 * ref_embs_norm, 0.3 * ref_phy_norm])

\# 测试集加权特征矩阵（用同样的权重）

test_weighted = np.hstack([0.7 * test_embs_norm, 0.3 * test_phy_norm])

pca = PCA(n_components=2)

ref_pca = pca.fit_transform(ref_weighted) # 参考集fit

test_pca = pca.transform(test_weighted)	# 测试集只transform

var_ratio = pca.explained_variance_ratio_

print(f"✅ PCA方差：PC1={var_ratio[0]*100:.2f}%, PC2={var_ratio[1]*100:.2f}%")

\# 保存PCA坐标

df_test_pca = pd.DataFrame(test_pca, index=test_pids, columns=["PC1","PC2"])

df_test_pca.to_csv(os.path.join(save_dir, "test_pca_coordinates.csv"), encoding='utf-8-sig')

\# ===================== 图4：测试集理化特征热图 =====================

plt.rcParams['font.sans-serif'] = ['Arial']

plt.rcParams['axes.unicode_minus'] = False

\# 用测试集自己的Z-score做热图（仅用于可视化，不影响分析）

test_phy_scaler = StandardScaler()

test_phy_scaled = test_phy_scaler.fit_transform(test_phy)

df_test_scaled = pd.DataFrame(test_phy_scaled, index=test_pids, columns=["MW","pI","GRAVY","Charge","Helix","Sheet"])

plt.figure(figsize=(10, 7))

sns.heatmap(df_test_scaled, cmap="viridis", annot=False,

xticklabels=True, yticklabels=True,

cbar_kws={'label': 'Standardized value (Z-score)'})

plt.title("Physicochemical Characteristics of 11 Test Proteins", fontsize=13, pad=12)

plt.xlabel("Physicochemical indices", fontsize=11)

plt.ylabel("Protein sequences", fontsize=11)

plt.xticks(rotation=45, ha='right', fontsize=9)

plt.yticks(rotation=0, fontsize=8)

plt.tight_layout()

plt.savefig(os.path.join(save_dir, "Fig4_physicochemical_heatmap_11test.png"), dpi=300, bbox_inches='tight')

plt.close()

print("✅ Fig4 已保存")

\# ===================== 图5：测试集融合相异度热图 =====================

plt.figure(figsize=(10, 8))

sns.heatmap(df_F_test, cmap="coolwarm", square=True, vmin=0, vmax=1,

cbar_kws={'label': 'Fused dissimilarity'})

plt.title("Fused Dissimilarity Matrix of 11 Test-set Proteins", fontsize=13, pad=12)

plt.xticks(rotation=45, ha='right', fontsize=8)

plt.yticks(rotation=0, fontsize=8)

plt.tight_layout()

plt.savefig(os.path.join(save_dir, "Fig5_fused_dissimilarity_heatmap_11test.png"), dpi=300, bbox_inches='tight')

plt.close()

print("✅ Fig5 已保存")

\# ===================== 图6：测试集PCA投影图 =====================

plt.figure(figsize=(10, 8))

\# 9条cathelicidin用蓝色，2条tick阳性对照用红色

cathelicidin_idx = [i for i, p in enumerate(test_pids) if p not in ['ABI52664.1', 'QOJ01963.1']]

tick_idx = [i for i, p in enumerate(test_pids) if p in ['ABI52664.1', 'QOJ01963.1']]

plt.scatter(test_pca[cathelicidin_idx, 0], test_pca[cathelicidin_idx, 1],

s=120, c='#2980b9', alpha=0.9, edgecolor='white', linewidth=0.6, label='Cathelicidin (n=9)')

plt.scatter(test_pca[tick_idx, 0], test_pca[tick_idx, 1],

s=150, c='#d63031', alpha=0.95, edgecolor='white', linewidth=0.8, marker='^', label='Tick Lipocalin positive control (n=2)')

\# 标注编号

for idx, (x, y) in enumerate(test_pca):

plt.text(x, y, str(idx+1), fontsize=8, ha='center', va='center', color='black')

plt.xlabel(f"PC1 ({var_ratio[0]*100:.2f}%)", fontsize=12)

plt.ylabel(f"PC2 ({var_ratio[1]*100:.2f}%)", fontsize=12)

plt.title("PCA Projection of 11 Test-set Proteins\n(Reference-fitted feature space, 0.7:0.3 weighted fusion)", fontsize=12, pad=12)

plt.legend(fontsize=10, loc='best')

plt.grid(alpha=0.25, linestyle='--', linewidth=0.5)

plt.gca().spines['top'].set_visible(False)

plt.gca().spines['right'].set_visible(False)

plt.tight_layout()

plt.savefig(os.path.join(save_dir, "Fig6_test_PCA_projection_11.png"), dpi=300, bbox_inches='tight')

plt.close()

print("✅ Fig6 已保存")

print("\n🎉 全部完成！3张图已保存到：", save_dir)
